# Observational and genetic evidence support a relationship between cardiac autonomic function and blood pressure

**DOI:** 10.3389/fcvm.2023.1187275

**Published:** 2023-06-19

**Authors:** Zekai Chen, Siqi Wang, Zhen He, Balewgizie S. Tegegne, Arie M. van Roon, Judith C. S. Holtjer, Pim van der Harst, Harold Snieder, Chris H. L. Thio

**Affiliations:** ^1^ Department of Epidemiology, University Medical Center Groningen, University of Groningen, Groningen, Netherlands; ^2^ Department of Neurology, Center for Statistical Genetics, Gertrude H. Sergievsky Center, Columbia University Medical Center, Columbia University, New York, NY, United States; ^3^ Department of Vascular Medicine, University Medical Center Groningen, University of Groningen, Groningen, Netherlands; ^4^ Department of Environmental Epidemiology, Institute for Risk Assessment Sciences (IRAS), Utrecht University, Utrecht, Netherlands; ^5^ Department of Cardiology, University Medical Center Groningen, University of Groningen, Groningen, Netherlands; ^6^ Division of Heart & Lungs, Department of Cardiology, Utrecht University Medical Center, Utrecht University, Utrecht, Netherlands

**Keywords:** cardiac autonomic function, heart rate, heart rate variability, blood pressure, two-sample Mendelian randomization

## Abstract

**Background:**

It is unclear how cardiac autonomic function, as indicated by heart rate (HR), heart rate variability (HRV), HR increase during exercise, and HR recovery after exercise, is related to blood pressure (BP). We aimed to examine the observational and genetic evidence for a potential causal effect of these HR(V) traits on BP.

**Methods:**

We performed multivariable adjusted linear regression using Lifelines and UK Biobank cohorts to investigate the relationship between HR(V) traits and BP. Linkage disequilibrium score regression was conducted to examine genetic correlations. We used two-sample Mendelian randomization (2SMR) to examine potential causal relations between HR(V) traits and BP.

**Results:**

Observational analyses showed negative associations of all HR(V) traits with BP, except for HR, which was positively associated. Genetic correlations were directionally consistent with the observational associations, but most significant genetic correlations between HR(V) traits and BP were limited to diastolic blood pressure (DBP). 2SMR analyses suggested a potentially causal relationship between HR(V) traits and DBP but not systolic blood pressure (SBP). No reverse effect of BP on HR(V) traits was found. One standard deviation (SD) unit increase in HR was associated with a 1.82 mmHg elevation of DBP. In contrast, one ln(ms) unit increase of the root mean square of the successive differences (RMSSD) and corrected RMSSD (RMSSDc), decreased DBP by 1.79 and 1.83 mmHg, respectively. For HR increase and HR recovery at 50 s, every additional SD increase was associated with a lower DBP by 2.05 and 1.47 mmHg, respectively. Results of secondary analyses with pulse pressure as outcome were inconsistent between observational and 2SMR analyses, as well as between HR(V) traits, and therefore inconclusive.

**Conclusion:**

Both observational and genetic evidence show strong associations between indices of cardiac autonomic function and DBP, suggesting that a larger relative contribution of the sympathetic versus the parasympathetic nervous system to cardiac function may cause elevated DBP.

## Introduction

Hypertension, characterized by chronically elevated blood pressure (BP), is a main risk factor for serious and disabling diseases and is responsible for an estimated 10 million deaths every year (1). The dysfunction of autonomic cardiovascular control has been long documented in the pathophysiology of hypertension, and its role in short-term BP regulation via the baroreflex is well recognized (2). Non-invasive clinical indicators of poorer cardiac autonomic function, including higher heart rate (HR), lower heart rate variability (HRV), delayed HR response to exercise, and delayed HR recovery after exercise, have all been shown to associate with elevated BP, as shown below.

The association between increased HR and elevated BP has long been observed (3, 4). The variation in beat-to-beat intervals as measured by HRV, is thought to reflect the effect of the sympathovagal balance (i.e., relative influence of the sympathetic nervous system, SNS, to that of the parasympathetic nervous system, PNS) on heart rhythm (5). In a longitudinal observational study with over 9 years of follow-up, compared with the highest quartile of HRV, the lowest quartile predicted higher risk of incident hypertension with increasing risk of 36% and 24% predicted by the root mean square of successive differences (RMSSD) and standard deviation of normal-to-normal R-R intervals (SDNN), respectively (6). HR increase in response to exercise and HR recovery post exercise represent distinct classical regulatory mechanisms of the cardiac autonomic nervous system (7). Delayed HR recovery post exercise was associated with attenuation of the nocturnal BP fall in both normotensive and hypertensive populations (8).

In addition to phenotypic evidence from observational studies, there is some genetic evidence for a relation between indicators of cardiac autonomic function and BP. There is a significant negative genetic association between HRV and BP, as was shown in a large community-based family study (9), which is consistent with the results of observational studies. Verweij and colleagues (10) constructed a polygenic score of HR response to exercise and found a strong association between the polygenic score and lower diastolic blood pressure (DBP). For HR, there was no evidence of a genetic relationship between HR and BP or hypertension (11).

Thus, many studies demonstrated links between markers of cardiac autonomic function and BP on the phenotypic and genetic level. However, it is uncertain whether cardiac autonomic function, as indicated by HRV and HR measures, is causally related to BP. In the current study, we aimed to strengthen the evidence for a potential causal effect of these HR(V) traits on BP using two-sample Mendelian randomization (2SMR), which utilizes genetic variants to strengthen causal inference and minimize bias in observational studies (12). In addition, we estimated phenotypic associations and genetic correlations in an effort to triangulate our findings (13).

## Methods

### Overall study design

**Figure 1:**
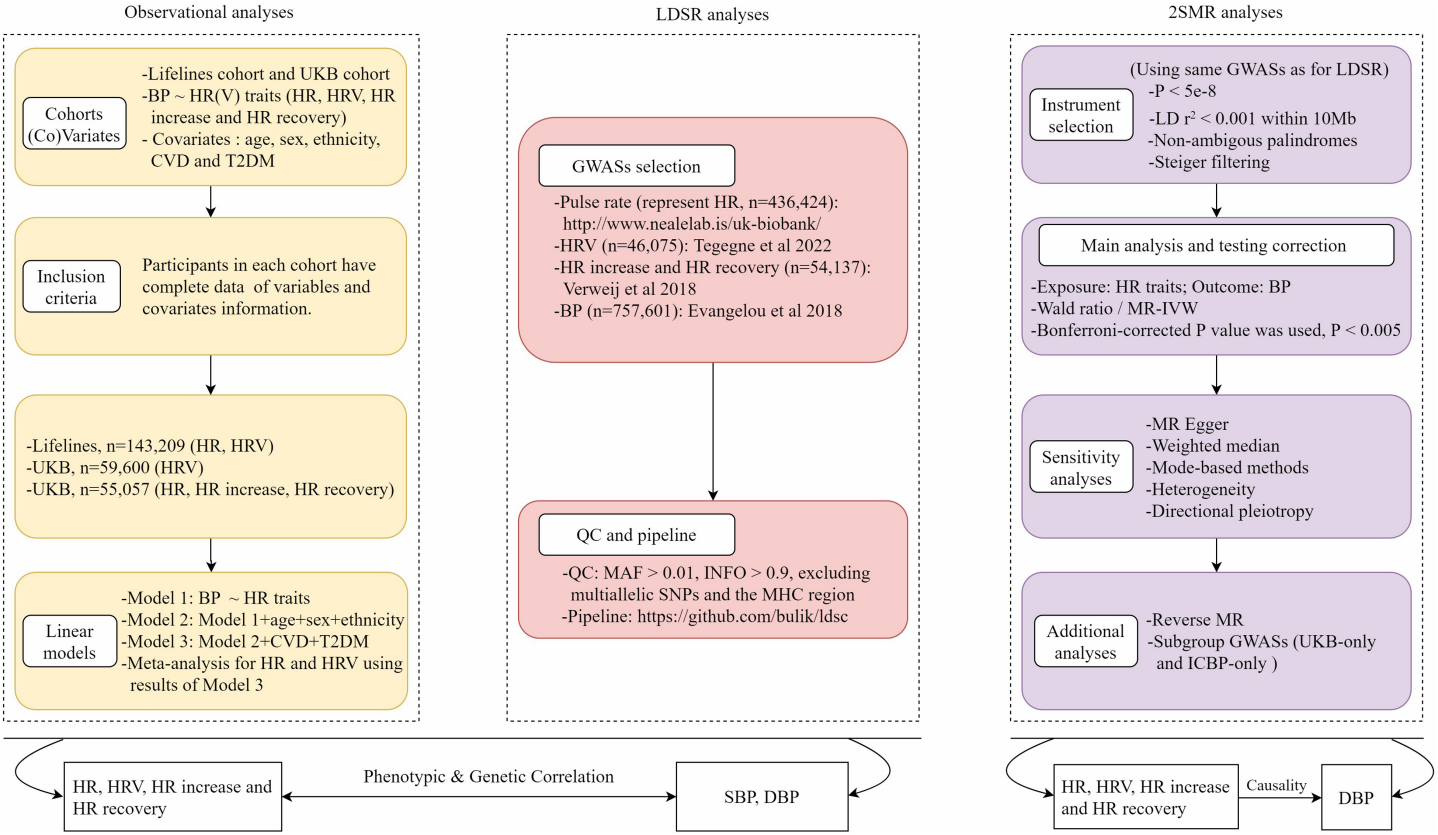
Overview of study design. HR, heart rate; HRV, heart rate variability; BP, blood pressure; LDSR, linkage disequilibrium score regression; MR, Mendelian randomization; UKB, UK Biobank; GWAS, genome-wide association study; IVs, instrumental variables.

This study consisted of three main parts, including (1) traditional observational analyses for which both data from the Lifelines Cohort Study and the UK Biobank (UKB) were used to investigate the phenotypic associations between HR(V) traits and BP; (2) linkage disequilibrium score regression (LDSR) analyses for which the latest publicly available genome-wide association study (GWAS) summary statistics for HR(V) phenotypes and BP were used to explore the genetic correlations; (3) 2SMR analyses for which the same GWAS summary data were used to determine the potential causal relationship between exposure and outcome. Overview of the study design can be seen in Figure 1.

### Observational analyses

#### Study population

Detailed description of Lifelines and UKB is provided in Supplementary Methods. Data of 143,209 participants from Lifelines was used to investigate the phenotypic relation of HR and HRV with BP. In UKB, 59,600 (for HRV) and 55,057 (for HR, HR response during and after exercise) participants were included in the observational analysis of HR(V) phenotypes and BP, respectively. Inclusion and exclusion criteria can be found in Supplementary Figure S1.

#### Definitions and measurements

In UKB, 3-lead (lead I, II, and III) electrocardiograms (ECG, AM-USB 6.5, Cardiosoft v6.51) at rest, during exercise and recovery post exercise were recorded from a sub-group of participants (*n* = 79,217, baseline visit) that underwent a cardio assessment using a stationary bicycle. From these ECGs, resting HR and HRV (15-second ECG), heart rate increase during exercise (6-min ECG) and heart rate recovery post exercise (1-min ECG) were calculated. In Lifelines, HR and HRV were obtained using the same calculation methods from the 10-second 12-lead resting ECG (Welch Allyn CardioPerfect software) results of ∼157k participants (14). In our study, HR, RMSSD, corrected RMSSD (RMSSDc), HR increase during exercise and HR recovery at 50 s after exercise were the primary HR(V) traits. More details on the measurement of HR(V) traits and BP, and on the definition of diseases (i.e., cardiovascular disease (CVD), type 2 diabetes (T2D) and hypertension) are given in the Supplementary Methods.

#### Statistical methods

Descriptive statistics and transformation of variable units are detailed in the Supplementary Methods. Multivariable linear regression models were performed using the Lifelines and UKB data to obtain observational estimates of the relationship between different HR(V) phenotypes and BP. In total, three models were applied as follows.

Model 1: BP∼HR(V) trait
Model 2: BP∼Model 1 + Age + Sex + Ethnicity
Model 3: BP∼Model 2 + CVD + T2D

Each model was applied for each individual HR(V) trait, and for systolic BP (SBP) and DBP separately. A directed acyclic graph was used to inform covariate selection (Supplementary Figure S2). In addition, we performed a random effects meta-analysis combining the results of Model 3 from Lifelines and UKB.

### Linkage disequilibrium score regression analyses

Table 1 includes details on the GWAS summary data used for LDSR. Detailed description of GWAS summary statistics can be found in Supplementary Methods. We implemented LDSR to estimate bivariate genetic correlations (15) between HR(V) traits and BP, using the open source *LDSC* software version 1.0.1 (https://github.com/bulik/ldsc) (16). More details can also be found in the Supplementary Methods.

**Table 1:** GWAS summary data used in two-sample Mendelian randomization and LD score regression.

Traits	Unit	Author	Year	Cohort/Consortium	Sample size	Population	No. of SNPs included	PMID/URL
Resting HR	SD	Elsworth	2018	UKB	436,424	European	272/264^a^	http://www.nealelab.is/uk-biobank/
lnRMSSD	ln(ms)	Tegegne	2021	UKB	46,075	European	14	https://doi.org/10.33612/diss.193633004
lnRMSSDc	ln(ms)	Tegegne	2021	UKB	46,075	European	11	
lnSDNN	ln(ms)	Tegegne	2021	UKB	46,075	European	6	
lnSDNNc	ln(ms)	Tegegne	2021	UKB	46,075	European	5	
HR increase	SD	Verweij	2018	UKB	54,137	European	11	29497042
HR increase	SD	Ramírez	2018	UKB	66,678	European	13	29769521
HR recovery (10 s)	SD	Verweij	2018	UKB	54,137	European	14	29497042
HR recovery (20 s)	SD	Verweij	2018	UKB	54,137	European	14	
HR recovery (30 s)	SD	Verweij	2018	UKB	54,137	European	17	
HR recovery (40 s)	SD	Verweij	2018	UKB	54,137	European	16	
HR recovery (50 s)	SD	Verweij	2018	UKB	54,137	European	15	
HR recovery (60 s)	SD	Ramírez	2018	UKB	66,678	European	15	29769521
BMI	kg/m^2^	Yengo	2018	UKB + GIANT	681,275	European	-	30124842
SBP	mmHg	Evangelou	2018	UKB + ICBP	757,601	European	-	30224653
DBP	mmHg	Evangelou	2018	UKB + ICBP	757,601	European	-	
PP	mmHg	Evangelou	2018	UKB + ICBP	757,601	European	-	

HR, heart rate; RMSSD, root mean square of successive differences; RMSSDc, corrected root mean square of successive differences; BMI, body mass index; SBP, systolic blood pressure; DBP, diastolic blood pressure; PP, pulse pressure; SD, standard deviation; ms, millisecond; kg/m^2^, kilogram/square meter; mmHg, millimeters of mercury; UKB, UK Biobank; GIANT, Genetic Investigation of ANthropometric Traits; ICBP, International Consortium of Blood Pressure; PMID, PubMed identity document; URL, uniform resource locator. SNPs finally included were with Steiger filtering after harmonizing.

^a^ When the outcome was SBP, 272 SNPs were included. When the outcome was DBP, 264 SNPs were included.

### Two-sample Mendelian randomization analyses

MR uses genetic variants as instrumental variables to strengthen causal inference from observational studies. Due to the fixed nature of genetic variants and Mendel's inheritance laws, estimates from MR are thought to be less sensitive to reverse causation and confounding (17). There are three key assumptions that need to be satisfied in MR (Supplementary Methods, Supplementary Figure S3). If these assumptions are met, associations derived from MR can be interpreted as causal effects.

Genome-wide significant SNPs (*p* < 5 × 10^−8^) associated with exposure [HR(V) traits] and outcome (BP) were selected from the most recent, comprehensive GWASs. Details of the GWAS datasets used in 2SMR are listed in Table 1. SNP instrument selection is detailed in Supplementary Methods.

Our main MR analysis was a random-effect inverse variance weighted (IVW) meta-analysis of individual SNP-specific Wald estimates. To assess violation of the exclusion restriction assumption due to horizontal pleiotropy, we performed diagnostic tests and adopted four complementary MR methods, namely MR Egger, weighted median, simple mode and weighted mode, that are pleiotropy-robust to varying degrees. Detailed description of the above methods is provided in Supplementary Methods.

The MR study followed the STROBE-MR checklist of recommended items to address in reports of Mendelian randomization studies (Supplementary Document S2). To account for multiple testing, a Bonferroni-corrected *P*-value significance threshold of <0.005 (0.05/10, five primary exposures and two outcomes) was determined to be statistically significant for all analyses (i.e., observational analysis, LDSR, and MR).

### Additional analyses

In the UK Biobank, we also performed multivariable linear regression models to obtain observational estimates of the relationships between pulse rate and BP. Considering the impact of beta-blockers on HR, we excluded beta-blocker users and re-estimated the observational relationships between HR and BP. Besides the primary HR(V) traits, analyses were also performed for SDNN, corrected SDNN (SDNNc), and HR recovery 10–40 s. We also utilized SNPs from Ramírez et al. (18) as instruments for HR increase and HR recovery (60 s) to validate the potential causal relationship with DBP, using the ICBP-only dataset. To test the robustness of our MR results to various sources of bias, we performed analyses using different datasets for BP, as well as multivariable MR (MVMR). We performed reverse MR to assess a potential reverse causal relationship [i.e., effect of BP on HR(V)]. Furthermore, we also examined pulse pressure (the difference between SBP and DBP) as an additional outcome. We performed multivariable linear regression models using both the Lifeline and UKB data to obtain observational estimates of the relationships between different HR(V) phenotypes and pulse pressure (PP). Using 2SMR, we aimed to explore the potential causal relationship between HR(V) traits and PP. More details are provided in the Supplementary Methods.

All analyses were performed using R software (version 3.6.2 and 4.0.3). All 2SMR analyses were conducted by using the *TwoSampleMR* (version 0.5.6) R package (19).

## Results

### Observational results

**Figure 2:**
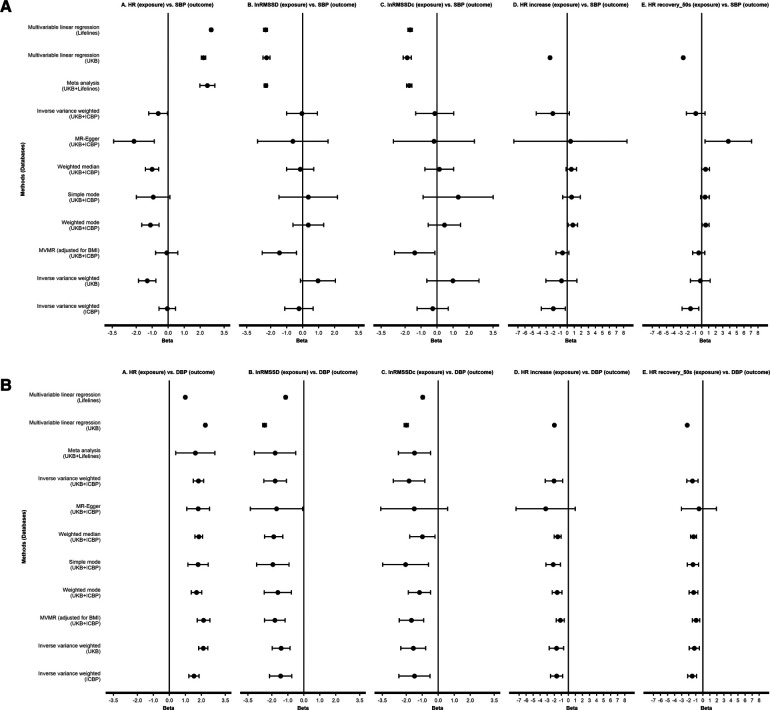
Observational analyses and Mendelian randomization analyses testing effect of HR(V) phenotypes on SBP (**A**) and DBP (**B**). All results are presented as estimates with 95% CIs. For linear regression and meta-analysis, results of model 3 are shown. HR, heart rate; RMSSD, root mean square of successive differences; RMSSDc, corrected root mean square of successive differences; SBP, systolic blood pressure; DBP, diastolic blood pressure; UKB, UK Biobank; ICBP, International Consortium of Blood Pressure.

Descriptives of Lifelines and UKB participants are shown in Supplementary Table S1. We found significant associations with SBP/DBP, where HR was positively correlated with BP, and other HR(V) traits were significantly and negatively correlated with BP (Figure 2, Supplementary Data S1). In Model 3 (our fully adjusted model), each SD unit higher HR, SBP was higher by respectively 2.69 mmHg (Lifelines), 2.21 mmHg (UKB), and 2.45 mmHg (meta-analysis). DBP was higher by 1.00 mmHg (Lifelines), 2.25 mmHg (UKB) and 1.62 mmHg (meta-analysis) with each SD unit higher HR. For RMSSD, each ln(ms) higher RMSSD, SBP was lower by respectively 2.32 mmHg (Lifelines), 2.26 mmHg (UKB), and 2.31 mmHg (meta-analysis) in Model 3. DBP was lower by 1.14 mmHg (Lifelines), 2.46 mmHg (UKB) and 1.80 mmHg (meta-analysis) with each ln(ms) unit higher RMSSD. Similar results were observed for RMSSDc, but the estimates were slightly smaller. During exercise, SBP and DBP were higher by 2.44 and 2.00 mmHg, respectively, with each SD unit higher HR. Each SD unit higher post-exercise HR recovery (50s), SBP and DBP were lower by 2.62 and 2.22 mmHg, respectively, in Model 3.

### LDSR results

**Figure 3:**
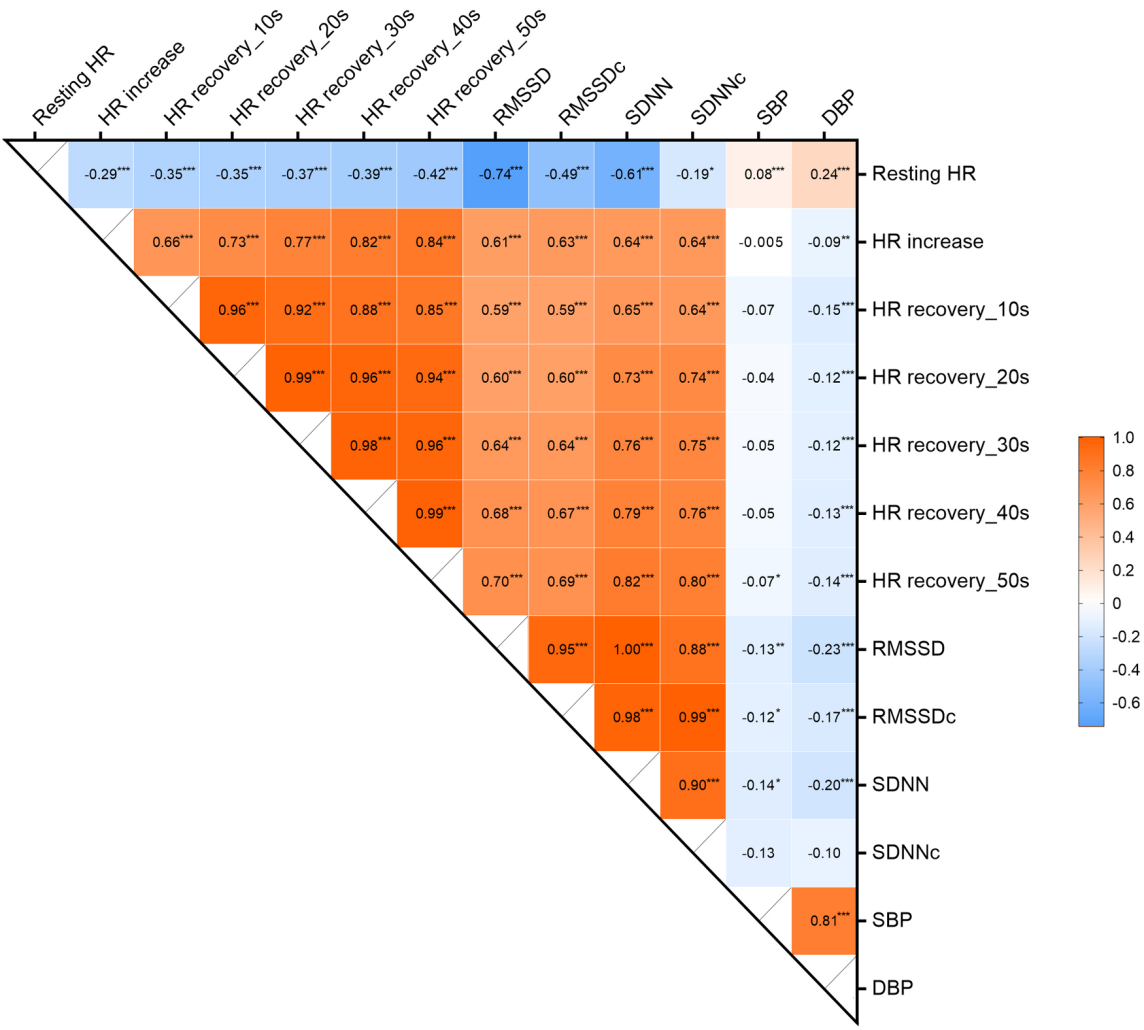
Genetic correlations between HR, HRV, HR increase, HR recovery and BP. ***p < 0.001, **p < 0.005, *p < 0.05. HR, heart rate; RMSSD, root mean square of successive differences; RMSSDc, corrected root mean square of successive differences; SDNN, normal-to-normal intervals; SDNNc, corrected normal-to-normal intervals; SBP, systolic blood pressure; DBP, diastolic blood pressure.

Between HR(V) traits, we found modest to near-perfect genetic correlations (*r*_g_ range 0.19–0.99). HR showed significant positive genetic correlations with SBP (*r*_g _= 0.08) and DBP (*r*_g _= 0.24) (Figure 3). The other HR(V) phenotypes had negative genetic correlations with BP, ranging from −0.005 to −0.14 for SBP and −0.09 to −0.23 for DBP. SBP only showed a significant genetic correlation with RMSSD (*r*_g _= −0.13). DBP had a low to moderate significantly negative correlation with all traits except SDNNc. The strongest negative correlation of DBP was with RMSSD (*r*_g _= −0.23).

### 2SMR results

Details of SNP instruments for HR(V) phenotypes are provided in Supplementary Results. Figure 2 shows the 2SMR results (more details in Supplementary Data S3). No robust effects were found between any HR(V) traits and SBP in the main analysis. Although suggestive evidence for a causal effect of resting heart rate on SBP was found, the main analysis [i.e., IVW with the combined UKB-ICBP (International Consortium of Blood Pressure) GWAS data] *P*-value did not survive our multiple testing threshold (*P *> 0.005). There were robust effects of each HR(V) trait on DBP. In several analyses, the MR Egger estimate was non-significant. However, we were reassured by the comparable effect magnitude from MR Egger, as well as the general absence of evidence for directional pleiotropy (MR Egger intercept *P* > 0.05, no influential SNPs, Supplementary Results).

One SD unit increase in HR was associated with a 1.82 mmHg elevation of DBP (95% CI 1.50–2.14, *P *= 2.17e-28). In contrast, per unit of ln(ms) increase of lnRMSSD and lnRMSSDc, DBP decreased by 1.79 mmHg (95% CI −2.50 to −1.08, *P* = 6.5e-7) and 1.83 mmHg (95% CI −2.82 to −0.84, *P* = 2.81e-4), respectively. For HR increase during exercise and HR recovery after exercise (50s), every SD increase was associated with a lower DBP of 2.05 mmHg (95% CI −3.27 to −0.82, *P* = 1.07e-3) and 1.47 mmHg (95% CI −2.27 to −0.68, *P* = 2.81e-4), respectively, (Figure 2; Supplementary Data S3).

### Additional results

A detailed description of the additional analyses results can be found in the Supplementary Results. Briefly, observational results showed similar estimates between pulse rate and BP comparable to HR in direction and magnitude in UKB. Excluding those using beta-blocker, we obtained results consistent with the main observational results, with both direction and magnitude being similar. Using other secondary HRV (i.e., SDNN and SDNNc) and HR recovery indicators (i.e., HR recovery 10–40 s), and using instrumental variables from another GWAS study on HR increase and HR recovery [Ramírez et al. (18)] yielded consistent estimates. Analyses in summary BP GWAS data from UKB and ICBP separately yielded results consistent with the main analysis, as were results from MVMR. Reverse MR showed little evidence of a causal effect of BP traits on HR(V) traits. In Lifelines, we found significant associations between HR and HRV with PP, but not in UKB, where significant negative associations were only found between HR increase, HR recovery (40 and 50 s) and PP. 2SMR showed potential causal effects of HR and HRV on PP, but no potential causal effects were found for other indicators of cardiac autonomic function.

## Discussion

The present study is the first to investigate the relation between cardiac autonomic function and BP using a combination of observational and genetic methods. Traditional observational analyses in UKB and Lifelines, LDSR, and 2SMR, converged on a potentially causal relationship of cardiac autonomic function indicators with DBP, but not with SBP. With each unit increase of these indicators of better cardiac autonomic function, DBP would be lower by approximately 2 mmHg, except for HR, which showed an expected positive causal effect on DBP. Meanwhile, little evidence of causal effects of BP on autonomic function indicators were found in reverse analysis, indicating unidirectional causal effects. Furthermore, the above results were corroborated by MR sensitivity analyses and additional analyses. Given these results, we propose that a larger relative contribution of the SNS versus the PNS to HR may cause elevated DBP.

As a surrogate for higher sympathetic activity (20–22), elevated HR has been repeatedly shown to be a risk factor for hypertension. Our study also obtained consistent results, where linear regression showed a positive relationship between HR and BP, and LDSR indicating the same trend, but the genetic correlation of HR with SBP was much weaker than that with DBP (0.08 vs. 0.24). Results from 2SMR suggest a potentially positive causal relationship appeared to exist only between HR and DBP and not SBP, which was also supported by sensitivity analysis. The results of both observational analysis and LDSR analysis of HRV are in agreement with previous studies. Schroeder et al. (6) found an inverse association between HRV and BP, and indicated the observational association between HRV and DBP was stronger than that of SBP, which is consistent with the study of Fagard and colleagues (23). We also found this in the LDSR results, where all HRV indicators (except for SDNNc) had stronger associations with DBP than with SBP. Tegegne et al. (9) used multiple publicly available GWAS summary statistics for LDSR analysis and also found that HRV (RMSSD and SDNN) had a greater negative genetic association with DBP than SBP. For the four HRV metrics, consistent results were found across almost all analyses, suggesting a potential causal relationship with DBP but not with SBP.

Mensah-Kane et al. (24) used 2SMR and found that HR increase during exercise and HR recovery post exercise have no clinically relevant effect on CVD; however, the study did not explore their potential causal associations with blood pressure. Our study confirmed for the first time that both HR increase during exercise and HR recovery after exercise were negatively related with BP at the phenotypic and genetic level. HR increase and HR recovery (10–50 s) showed statistically significant negative genetic correlations with DBP, but not with SBP, consistent with the negative relations estimated in the MR analyses. In the present study, we treated heart rate recovery as an indicator of cardiac autonomic function. However, heart rate recovery is likely to be affected by physical exercise. Previous research suggests that for patients with impaired cardiac autonomic function, exercise training can improve abnormal HR recovery (25, 26). Exercise training was found to associate with a relative enhancement of vagal tone, an improvement of HR recovery post-exercise, and a decrease in morbidity among patients with cardiovascular conditions (27). Abnormal HR recovery has been found to associate with all-cause mortality, while patients who normalized their HR recovery after completing phase 2 cardiac rehabilitation had survival rates similar to those with normal HR recovery (28). These studies highlight the importance of exercise training in improving heart rate recovery. Future study may disentangle the effects of heart rate recovery on cardiovascular health from those of physical activity.

Our findings suggest that increased HR and HRV cause lower and higher PP, respectively, which is contrary to our expectation that a larger contribution of the SNS versus PNS to cardiac function (i.e., higher HR and lower HRV) leads to less favourable outcomes (i.e., higher PP). We struggle to interpret this physiologically, but one possible explanation is that PP is calculated as a linear combination of SBP and DBP (i.e., SBP minus DBP). In 2SMR, we found evidence for a lowering effect of HRV on DBP but a null-effect on SBP, which could explain the association between higher HRV and PP. However, not all cardiac autonomic function indicators were associated with PP in 2SMR, and observational results in UKB and Lifelines did not converge with the 2SMR results. We therefore remain cautious in inferring causality between cardiac autonomic function and PP.

As one of the main mechanisms involved in the regulation of the cardiovascular system, cardiac autonomic function plays an important role in the development and progression of cardiovascular diseases such as hypertension, arrhythmia, and sudden cardiac arrest (29, 30). Due to the anatomical location of the cardiac autonomic nervous system and its complex function and distribution characteristics, it cannot be measured and evaluated simply and directly. Therefore, whether for clinical or scientific research, it is important to use sensitive, accurate and non-invasive indicators to represent cardiac autonomic function. The indicators we studied have been widely proven to be practical clinical indicators that represent cardiac autonomic function. Among these, elevated HR stands for relatively higher sympathetic activation (20–22), HRV represents sympathovagal control of the heart rhythm (5), and the response of heart rate to exercise reflects the process of alternating balance and coordination of the SNS and the PNS (7). During exercise, the cardiac autonomic system is characterized by higher activation of sympathetic nerves and a decrease in vagal tone, resulting in increased HR. After exercise, the opposite ensues and HR recovers.

Our study suggests that indicators of cardiac autonomic function are related to DBP but not SBP. There was no reverse effect of BP on HR(V) traits, suggesting unidirectionality. MR suggests that resting HR has a positive relationship with DBP, suggesting that a relatively higher activation of the cardiac sympathetic system in the resting state may lead to an increase in DBP. HRV and HR response to exercise were shown to have a negative relationship with DBP. Taken together, a relative shift towards a less favorable sympathovagal balance (i.e., relatively higher sympathetic tone) regulating cardiac rhythm, both in rest and in response to exercise, may cause higher DBP. There may be two explanations why a larger relative contribution to the HR of the SNS versus the PNS causes elevated DBP but not elevated SBP. First, sympathetic activation leads to higher systemic vascular resistance and, therefore, higher mean arterial pressure (MAP), which is dominated by DBP (MAP ≈ 2/3DBP + 1/3SBP). A second explanation is that SBP is influenced, more than DBP, by short term variations in HRV dampening fluctuations in BP through the baroreceptor reflex, which reduces the long term (causal) correlation (31).

Our study has several implications. It provides evidence of a potential causal effect of cardiac autonomic function on DBP, warranting future etiological investigation on the underlying biological mechanisms. Our study was based on data from the relatively healthy, general population. Therefore, translation to clinical practice is uncertain. An objective for future research could be to investigate the role of autonomic function in hypertensive patients with a predominantly elevated DBP; autonomic function is possibly a suitable treatment target, although our effect estimates predict a modest treatment effect. With regards to prevention, clinicians may consider monitoring indices of cardiac autonomic function to identify those at higher risk of developing hypertension.

Our study has several strengths. We used two large samples of different populations for the traditional observational analyses, and provide meta-analytic results. Similarly, GWAS studies used in our study also have (very) large sample sizes, allowing for the identification of multiple strong genetic instruments and precise estimation of their effects. Furthermore, we employed different approaches (i.e., traditional linear regression, LDSR and 2SMR) and different datasets to address distinct sources of bias. In addition we used a wide range of cardiac autonomic indices. Several limitations of our study should be noted. Firstly, in the main analysis of the 2SMR, we used Evangelou's BP GWAS summary statistics, which included data from UKB and the ICBP consortium (32). The partial sample overlap due to the involvement of UKB in both exposure and outcome GWAS may have introduced bias in our 2SMR estimates (33). Secondly, the UKB + ICBP GWAS, as well as the ICBP-only GWAS, adjusted for BMI, which could lead to collider bias (34). The UKB-only GWAS however was not adjusted for BMI. We therefore consider the UKB-only and ICBP-only GWAS summary data to vary in their potential sources of bias, and decided to perform 2SMR sensitivity analyses on the UKB-only and ICBP-only GWAS summary data. In addition, we used MVMR, in which we modelled BMI as a covariate; recently this has been shown to reduce potential collider bias due to GWAS adjustment for a heritable covariate (35). Reassuringly, each of these additional analyses yielded consistent results. Thirdly, observational and GWAS data used in the current study were predominantly derived from European populations, which limits the generalizability to other ethnicities and populations. Fourthly, currently available studies and data on the genetics of cardiac autonomic function metrics are limited with regards to sample size. Future larger studies may identify more genetic markers that can be used as instrumental variables for cardiac autonomic function and improve on the precision of our study. Finally, although our study provides robust and converging evidence, functional, experimental work is needed to establish causality more definitively between cardiac autonomic function and DBP.

## Conclusion

Both observational and genetic analyses show robust associations between indices of cardiac autonomic function and DBP, suggesting that a larger relative contribution of the SNS versus the PNS to heart rate may cause higher DBP but not higher SBP.

## Data Availability

The original contributions presented in the study are included in the article/supplementary material, further inquiries can be directed to the corresponding author/s. UKB and Lifelines data are not publicly available and can be obtained from a third party. Researchers interested in using the data from this study can apply for access through the respective websites: UKB (https://www.ukbiobank.ac.uk/enable-your-research/apply-for-access) and Lifelines (https://www.lifelines.nl/researcher/how-to-apply).
