# Supplementary material for: Observational and genetic evidence support a relationship between cardiac autonomic function and blood pressure
Source: Front Cardiovasc Med. 2023 Jun 19;10:1187275. doi: 10.3389/fcvm.2023.1187275 (PMC10315649; doi:10.3389/fcvm.2023.1187275)
Supplement: Supplementary file 1 [file Table1.docx]

**Observational and genetic evidence support a relationship between cardiac autonomic function and blood pressure**

Supplementary Material

Contents

Supplementary Data S1-S10 (.xlsx)

- Data S1. The results of linear regression models using both Lifelines and UK Biobank observational cohorts (BP)

- Data S2. SNPs used as IVs in MR analyses testing effect of HR(V) traits on BP (ID: ieu-b-38/39)

- Data S3. The effects of components of cardiac autonomic function on blood pressure using Mendelian randomization (Steiger filtering)

- Data S4. The results of heterogeneity analyses and the pleiotropy analyses of bi-directional Mendelian randomization

- Data S5. The effects of blood pressure on components of cardiac autonomic function using Mendelian randomization (Steiger filtering)

- Data S6. The results of linear regression models using both Lifelines and UK Biobank observational cohorts (PP)

- Data S7. The results of linear regression models using UK Biobank observational cohorts for pulse rate

- Data S8. The results of linear regression models for heart rate using Lifelines observational cohort (excluding beta-blocker users)

- Data S9. The effects of components of cardiac autonomic function on pulse pressure using Mendelian randomization (Steiger filtering)

- Data S10. The effects of components of cardiac autonomic function on DBP using Mendelian randomization (IVs from Ramírez et al.)

Supplementary Methods

Supplementary Results

Supplementary Table S1. Baseline characteristics of observational study participants from two cohorts

Supplementary Figure S1. Inclusion and exclusion criteria of traditional observational studies

Supplementary Figure S2. Directed acyclic graph of variable relationships for the traditional observational analysis

Supplementary Figure S3. Assumptions and rationale of two-sample Mendelian randomization

Supplementary Figure S4. Mendelian randomization scatterplots of HR(V) traits to blood pressure

Supplementary Figure S5. Mendelian randomization single SNP forest plots and leave-one-out plots

Supplementary Document 1. The list of all ICBP authors from the 77 ICBP studies contributing to the ICBP meta-analysis

Supplementary Document 2. STROBE-MR checklist of recommended items to address in reports of Mendelian Randomization studies

Supplementary References

**Supplementary Methods**

## **Observational analyses**

#### *Study population*

Both Lifelines and UKB are prospective, large population-based cohort studies, recruiting from 2006. Lifelines is a multi-disciplinary prospective population-based cohort study examining in a unique three-generation design the health and health-related behaviours of 167,729 persons living in the North of the Netherlands. It employs a broad range of investigative procedures in assessing the biomedical, socio-demographic, behavioural, physical and psychological factors which contribute to the health and disease of the general population, with a special focus on multi-morbidity and complex genetics. The UKB study recruited half a million individuals from the UK with an age range of 40 to 69 years and have performed in-depth data collection, including a broad range of environmental exposures and phenotypes in conjunction with genetic data. The UKB and Lifelines were approved by the North West Multi‐Centre Research Ethics Committee and the University Medical Center Groningen Ethics Committee, respectively. Written informed consent was obtained from all participants. Detailed study design and methods used by UKB^(1)^ and Lifelines^(2)^ have been described elsewhere.

#### *Definitions and measurements*

In UKB, 3-lead (lead I, II, and III) electrocardiograms (ECG, AM-USB 6.5, Cardiosoft v6.51) at rest (15 seconds), during exercise (6 minutes) and recovery post exercise (1minute) were recorded from a sub-group of participants (n=79,217, baseline visit) that underwent a cardio assessment (50% or 30% of maximum exercise load) using a stationary bicycle. More details on the exercise protocol and screening process for the cardio assessment can be elsewhere^(3)-(4)^. HRV was calculated based on the variation in inter-beat interval, using previously developed dedicated software^(5)^ to detect R-peaks. RMSSD and corrected RMSSD (RMSSDc), which corrected RMSSD for its dependency on HR^(6)^, were estimated as indices of HRV. SDNN and heart rate corrected SDNN (SDNNc), were also calculated as indices of HRV. HR increase in response to exercise was defined as the difference between maximum HR during exercise and resting HR. HR recovery post exercise was determined as the difference between peak HR during exercise and mean HR at 10, 20, 30, 40 and 50 seconds after the exercise had stopped.

In UKB, BP (and pulse rate) was measured using the Omron HEM 7015-T digital blood pressure monitor (Omron Healthcare) and the average of the two repeated automated measurements in mmHg (and beats/min) was used. In 2SMR, we used pulse rate to represent resting HR, which had the largest GWAS sample size, to extract instrumental variables for HR. In Lifelines, a DinaMap PRO 100V2 (GE Healthcare, Freiburg, Germany) was used to measure BP, every minute for a total of ten minutes; the mean of the last three measurements in mmHg was used. To account for the effect of medication use, +15 and +10 mmHg were added for systolic blood pressure (SBP) and DBP, respectively, among participants using antihypertensive medication^(7)^. This method has been shown to reduce bias and improve statistical power^(7)^.

Cardiovascular diseases (CVD), including myocardial infarction, stroke, arrhythmia, and other vascular diseases, were defined based on ICD codes (ICD-10 for Lifelines, ICD-9, ICD-10 and OPCS-4 for UKB) and questionnaire (self-reported CVD and related symptoms)^(8)-(9)^. In Lifelines, type 2 diabetes (T2D) was defined based on either self-reported T2D, use of anti-T2D medication, fasting plasma glucose >= 7.0 mmol/L, or HbA1c>=6.5%^(10)^. In UKB, T2D was defined based on ICD-9, ICD-10and self-reported questionnaire^(11)^. Hypertension was defined as a SBP >= 140 mmHg, or DBP >= 90 mmHg, or taking antihypertensive drugs^(12)^.

#### *Statistical methods*

For descriptive statistics, continuous variables that conformed to a normal distribution were presented as mean ± standard deviation. Data with a non-normal distribution were expressed as median (interquartile range). Categorical variables were described as the number of samples (percentage). The HRV indicators were transformed by the natural logarithm (ln) to obtain an approximately normal distribution. Consistent with the exposure units used in 2SMR, we standardized the other HR(V) traits (i.e. HR, HR response during and after exercise) so that each had a mean of 0 and a variance of 1 using an inverse rank normal transformation.

#### Linkage disequilibrium score regression analyses

We used GWAS summary statistics from UKB for HRV (n=46,075)^(13)^, resting HR (n=436,424)^(14)^, HR increase in response to exercise (n=54,137)^(3)^ and HR response to recovery post exercise (n=54,137)^(3)^. BP summary-level data from a recently conducted GWAS^(15)^ which incorporated a two-stage design with a total of 757,601 participants from the UKB and the International Consortium of Blood Pressure (ICBP) were used as outcome data in our study. ICBP GWAS, an international consortium exploring blood pressure genetics, was used by Evangelou et al. and consisted of a GWAS dataset from 77 cohorts containing 299k participants^(15)^.

The 1000 Genomes Project reference panel of European ancestry was used for linkage disequilibrium (LD) between single nucleotide polymorphisms (SNP). We selected well-imputed HapMap3 SNPs. SNPs within the major histocompatibility complex region were excluded from the analysis to avoid pleiotropic effects^(16)^. Moreover, SNPs were further filtered by minor allele frequency > 0.01, and an imputation score INFO > 0.9 when this information was available in the summary dataset.

## **Two-sample Mendelian Randomization analyses**

#### *Assumptions*

The first assumption is the relevance assumption, that is, the existence of a strong, robust relationship between the genetic variant and exposure (HR(V) phenotypes) is required. Second is the independence assumption, which supposes there is no association between the genetic variant and any confounders in the relationship between exposure and outcome. This second assumption is supported by the random allocation and independent assortment of genetic variants at conception. Third is the exclusion restriction assumption that the genetic variant is assumed to influence the outcome (BP) only through the exposure variable, and not through other, horizontally pleiotropic, pathways. As this third assumption is untestable, we performed strict instrument selection and performed a range of MR sensitivity analyses robust to varying degrees of pleiotropy (detailed below).

#### *Genetic instruments for 2SMR*

The exposure data was clumped with a clumping window of 10 Mb and LD r^2^ < 0.001 to guarantee independent genetic variants. For HRV, we directly used the genome-wide significant variants recently discovered by Tegegne et al.^(13)^ If a SNP instrument for the HR(V) phenotypes was not available in the outcome GWAS dataset, it was replaced with a proxy SNP (LD r^2^>0.8 according to the European 1000 Genomes Project reference panel) or removed in the absence of such a proxy. We harmonized SNP allele between exposure and outcome datasets and removed palindromic SNPs with intermediate allele frequencies (0.42-0.58). The Steiger filtering method^(17)^ was applied to remove potentially invalid instrumental variables that explain more variance in BP than in the HR(V) phenotypes.

#### *2SMR methods and sensitivity analyses*

IVW assumes that 100% of genetic variants are valid instrumental variables^(18)^, and the weighted median assumes at least 50% and estimates the effect based on the median SNP^(19)^. As long as the pleiotropic effects are independent of instrument strength (InSIDE assumption), the MR Egger can provide a consistent estimate even if all the genetic variants have pleiotropic effects^(20)^. The mode-based models assume that the most common causal effect estimator represents the true causal effect on the outcome. The difference between weighted mode and simple mode is the weight assigned to each ratio estimate^(21)^.

Diagnostic tests including heterogeneity tests using IVW and MR Egger, and the Egger intercept pleiotropy test were performed^(22)^. In case the *P* for the MR Egger intercept was smaller than 0.05, which is indicative of directional pleiotropy, the IVW estimate was likely biased and pleiotropy-robust MR sensitivity analyses (e.g. MR Egger) were closely examined for consistency with IVW. Furthermore, single SNP effects, leave-one-SNP-out analysis, and MR scatterplots, were conducted to visually examine heterogeneity and potential directional pleiotropy.

Additional analysesDue to potential bias introduced by overlap in participants between the exposure and outcome GWAS samples (i.e. each HR(V) phenotype vs. BP), and body mass index (BMI) adjustment in the BP GWAS, we additionally used summary GWAS of BP from UKB^(14)^ and ICBP^(15)^ separately to further validate our results^(23)-(24)^. When the BP summary statistics from UKB were used, BMI was not adjusted for, but there was still the issue of sample overlap. When using summary data from ICBP, the two samples did not overlap, but BMI adjustment in this GWAS potentially induced collider bias. To correct for potential collider bias from BMI adjustment, we performed sensitivity analysis in which BMI was included as a covariate in multivariable MR (MVMR)^(25)^.

We also performed reverse MR, which explored the potentially causal effects of BP on HR(V) traits to determine whether there was a reverse causal relationship. In this case, the same procedure was followed as for the 2SMR mentioned above, except BP was used as the exposure and HR(V) phenotypes as the outcome.

**Supplementary Results**

#### 2SMR analyses

For our 2SMR analyses, we used 272/264 (corresponding to SBP/DBP) SNP instruments for HR, 14 for lnRMSSD, 11 for lnRMSSDc, 6 for lnSDNN, 5 for lnSDNNc, 11 for HR increase, 14, 14, 17, 16 and 15 respectively for HR recovery at 10, 20, 30, 40 and 50 seconds. Supplementary Data S2 shows details on the SNPs, including information on position, alleles, and effects. F-statistics for each SNP indicated sufficient instrument strength (F>10) ^(26)-(27)^.

Supplementary Data S4 shows the results of heterogeneity and pleiotropy analyses for the 2SMR analyses. There was significant heterogeneity, implying potential pleiotropy. However, there was little evidence of directional pleiotropy. In addition to pleiotropy analyses, we used MR scatterplots (Supplementary Figure S4), as well single SNP forest plots and leave-one-out plots to visually examine potential horizontal pleiotropy (Supplementary Figure S5). Although these plots showed considerable heterogeneity (i.e. widely varying single SNP Wald ratios), we were reassured by the lack of evidence of directional pleiotropy and overly influential, potentially pleiotropic SNPs.

## **Additional analyses**

In UKB, observational results showed similar estimates between pulse rate and BP comparable to HR in direction and magnitude (Supplementary Data S7**)**. In Model 3, each SD unit higher pulse rate, SBP and DBP were higher by 2.04 and 2.38 mmHg, respectively. Excluding those using beta-blocker, we obtained consistent results with both direction and magnitude being similar (Supplementary Data S8**)**. In Model 3, each SD unit higher HR, SBP was higher by respectively 3.06 mmHg (Lifelines), 2.56 mmHg (UKB), and 2.81 mmHg (meta-analysis). DBP was higher by 1.16 mmHg (Lifelines), 2.42 mmHg (UKB) and 1.79 mmHg (meta-analysis) with each SD unit higher HR.

Other indices of HRV, namely SDNN and SDNNc, showed an effect on DBP but not on SBP. MR analyses yielded similar estimates between SDNN(c) and DBP comparable to RMSSD(c) in direction and magnitude. Except for HR recovery at 10s, all post-exercise HR recovery phenotypes yielded results similar to HR recovery at 50s. HR recovery at 10s had no significant effects on either SBP or DBP (Supplementary Data S3). Employing the instrumental variables from Ramírez et al. for HR increase and HR recovery (60s), we obtained consistent estimates on DBP in ICBP (Supplementary Data S10). For HR increase during exercise and HR recovery after exercise (60s), every SD increase was associated with a lower DBP of 1.45 mmHg (95%CI -2.68 to -0.23, P = 1.99e-2) and 1.50 mmHg (95%CI -2.45 to -0.56, P = 1.73e-3), respectively (Supplementary Data S10).

For summary GWAS data of BP from UKB and ICBP alone, the results exhibited results consistent with the combined database (i.e. UKB+ICBP). Except for HR, none of the exposure phenotypes were associated with SBP either in the UKB-only or ICBP-only data. HR and SBP only exhibited a significant association in the UKB database (beta=-1.31, P= 2.25e-6), but in the opposite direction to the linear regression and LDSR results, with potential bias due to directional pleiotropy (MR-Egger intercept=0.04, *P* = 1.03e-03). This result was also not supported by the primary analysis (i.e. using the UKB+ICBP database) nor by the separate ICBP-only result, which cast doubt on the results. For DBP, both UKB and ICBP data separately showed results consistent with the main analysis in direction and magnitude, indicating minimal bias due to sample overlap and/or BMI-adjustment. MVMR results were also consistent with the main analysis suggesting little bias due to GWAS adjustment for BMI (Figure 2; Supplementary Data S3).

Reverse MR, i.e. the causal effect of BP traits on HR(V) traits, was performed to exclude a bidirectional causal relationship (Supplementary Data S5). The reverse MR analyses did not suggest potential causal effects of SBP on HR, RMSSD, RMSSDc, HR increase and HR recovery. In the combined UKB+ICBP dataset, DBP was found to have significant effects on RMSSDc. However, this reverse effect was not supported by sensitivity analyses, indicating these results were likely driven by pleiotropic SNPs. There were no significant effects of DBP on other primary HR(V) traits.

Observational results of relationship between different HR(V) phenotypes and PP in Lifelines and UKB cohorts are shown in Supplementary Data S6. In Lifelines, we found significant associations with PP, where HR was positively correlated with PP, and all HRV traits were significantly and negatively correlated with PP. In Model 3, for each SD unit higher HR, PP was higher by 1.69 mmHg. For HRV, with each ln(ms) higher RMSSD(c) and SDNN(c), PP was lower by respectively 1.18 (0.70) mmHg and 1.07 (0.40) mmHg in Model 3. In UKB, significant associations were only found between HR increase, HR recovery (40s and 50s) and PP. In Model 3, for each SD higher in HR during exercise, PP was lower by 0.44 mmHg. For each SD unit higher post-exercise HR recovery (40s) and HR recovery (50s), PP was lower by 0.22 and 0.39 mmHg for PP, respectively, in Model 3. For the meta-analysis, significant association was only found between SDNNc and PP, and PP was lower by 0.33 mmHg with each ln(ms) higher SDNNc.

2SMR results between different HR(V) traits and PP are shown in Supplementary Data S9. One SD unit increase in HR was associated with a 2.61 mmHg decrease of PP (95%CI -2.97 to -2.25, P = 3.37e-46). Per unit of ln(ms) increase of RMSSD and SDNN, PP increased by 1.82 mmHg (95%CI 0.99 to 2.65, P = 1.85e-5) and 2.82 mmHg (95%CI 1.61 to 4.02, P = 4.62e-6), respectively, comparable to the RMSSDc and SDNNc estimates. No robust effects of HR increase and HR recovery on PP were found.

Despite significant heterogeneity in the HR(V)-PP analyses, there was little evidence of bias due to directional pleiotropy, except for a potential underestimation of the HR-PP relation by the IVW method (Supplementary Data S9).

**Supplementary Table S1. Baseline characteristics of study participants in Lifelines and UKB**

| **Characteristics** | **Lifelines**  **(n=143,209)** | **UKB for HRV**  **(n=59,600)** | **UKB for HR response to exercise**  **(n=55,057)** |
| --- | --- | --- | --- |
| Age (years), mean (SD) | 44.54 (12.92) | 57.30 (8.03) | 57.10 (8.11) |
| Male, n (%) | 59,232 (41.36%) | 26,971 (45.25%) | 25,719 (46.71%) |
| HR (bpm), mean (SD) | 67.36 (11.12) | - | 71.76 (11.73) |
| RMSSD (ln(ms)), mean (SD) | 3.34 (0.72) | 3.03 (0.65) | - |
| RMSSDc (ln(ms)), mean (SD) | 1.14 (0.66) | 0.88 (0.58) | - |
| SDNN (ln(ms)), mean (SD), | 3.36 (0.67) | 3.27 (0.61) | - |
| SDNNc (ln(ms)), mean (SD), | 1.15 (0.63) | 1.12 (0.58) | - |
| SBP (mmHg), mean (SD) | 125.34 (15.20) | 132.46 (17.57) | 131.93 (16.52) |
| DBP (mmHg), mean (SD) | 73.64 (9.30) | 81.64 (8.30) | 81.61 (7.99) |
| PP (mmHg), mean (SD) | 51.70 (11.13) | 50.81 (13.01) | 50.32 (12.25) |
| SBP^#^ (mmHg), mean (SD) | 127.14 (16.91) | 135.69 (19.79) | 134.99 (18.77) |
| DBP^#^ (mmHg), mean (SD) | 74.84 (10.25) | 83.80 (9.67) | 83.65 (9.38) |
| PP^#^ (mmHg), mean (SD) | 52.30 (11.53) | 51.89 (13.59) | 51.34 (12.83) |
| CVD, n (%) | 14,940 (10.44%) | 8,932 (14.99%) | 7,324 (13.30%) |
| T2DM, n (%) | 4,313 (3.02%) | 2,304 (3.87%) | 1,933 (3.51%) |
| Hypertension, n (%) | 36,568 (25.53%) | 17,925 (30.08%) | 15,557 (28.26%) |
| Antihypertensive medication use, n (%) | 17,254(12.16%) | 12,832 (21.53%) | 11,244 (20.42%) |
| HR increase (bmp), mean (SD) | - | - | 45.31 (13.06) |
| HR recovery at 10s (bpm), mean (SD) | - | - | 17.92 (7.68) |
| HR recovery at 20s (bpm), mean (SD) | - | - | 23.35 (8.98) |
| HR recovery at 30s (bpm), mean (SD) | - | - | 27.38 (9.78) |
| HR recovery at 40s (bpm), mean (SD) | - | - | 30.45 (10.32) |
| HR recovery at 50s (bpm), mean (SD) | - | - | 32.66 (10.74) |
| Abbreviations: HR, heart rate; HR, heart rate variability; RMSSD, root mean square of successive differences; RMSSDc, corrected root mean square of successive differences; SDNN, standard deviation of normal-to-normal R-R intervals; SDNNc, corrected standard deviation of normal-to-normal R-R intervals; SBP, systolic blood pressure; DBP, diastolic blood pressure; CVD: cardiovascular disease; T2DM, type 2 diabetes mellitus; UKB, UK Biobank; SD, standard deviation.  # To adjust for the effect of antihypertensive medication, +15, +10 mmHg and +5 mmHg were added for SBP, DBP and PP, respectively. | | | |

**Supplementary Figure S1. Inclusion and exclusion criteria for the traditional observational study samples**


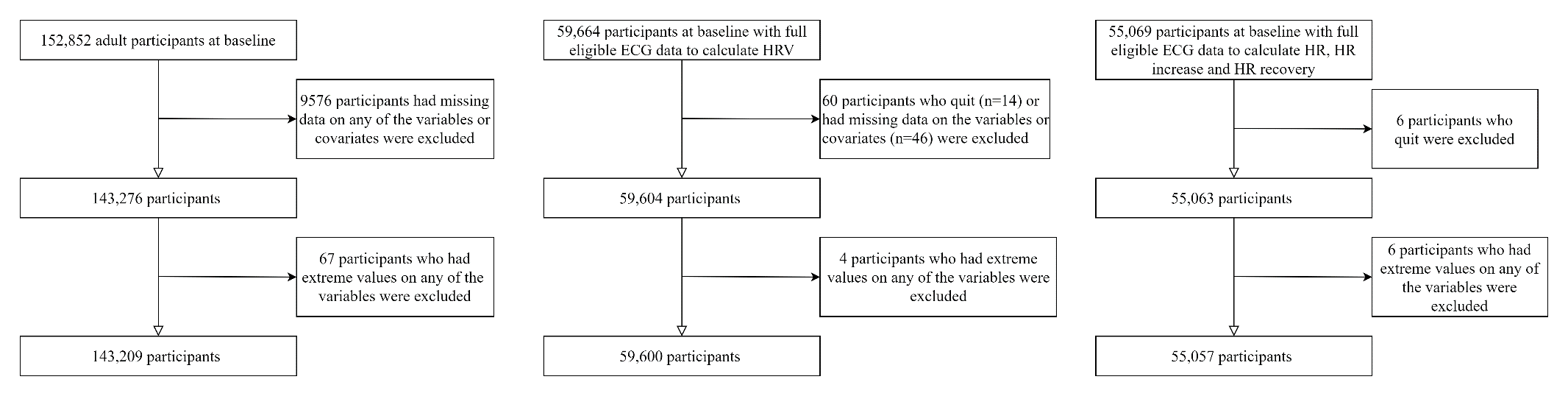


Supplementary Figure S1a Supplementary Figure S1b Supplementary Figure S1c

Supplementary Figure S1a. Flowchart of the included adult participants in Lifelines (for HR and HRV).

Supplementary Figure S1b. Flowchart of the included participants in UK Biobank (for HRV).

Supplementary Figure S1c. Flowchart of the included participants in UK Biobank (for HR, HR increase during exercise and HR recovery after exercise).

extreme values refer to higher or lower than 5 standard deviations from the mean value.

Abbreviations: HR, heart rate; HRV, heart rate variability; ECG, electrocardiogram.

**Supplementary Figure S2. Directed acyclic graph of variable relationships for the traditional observational analysis**


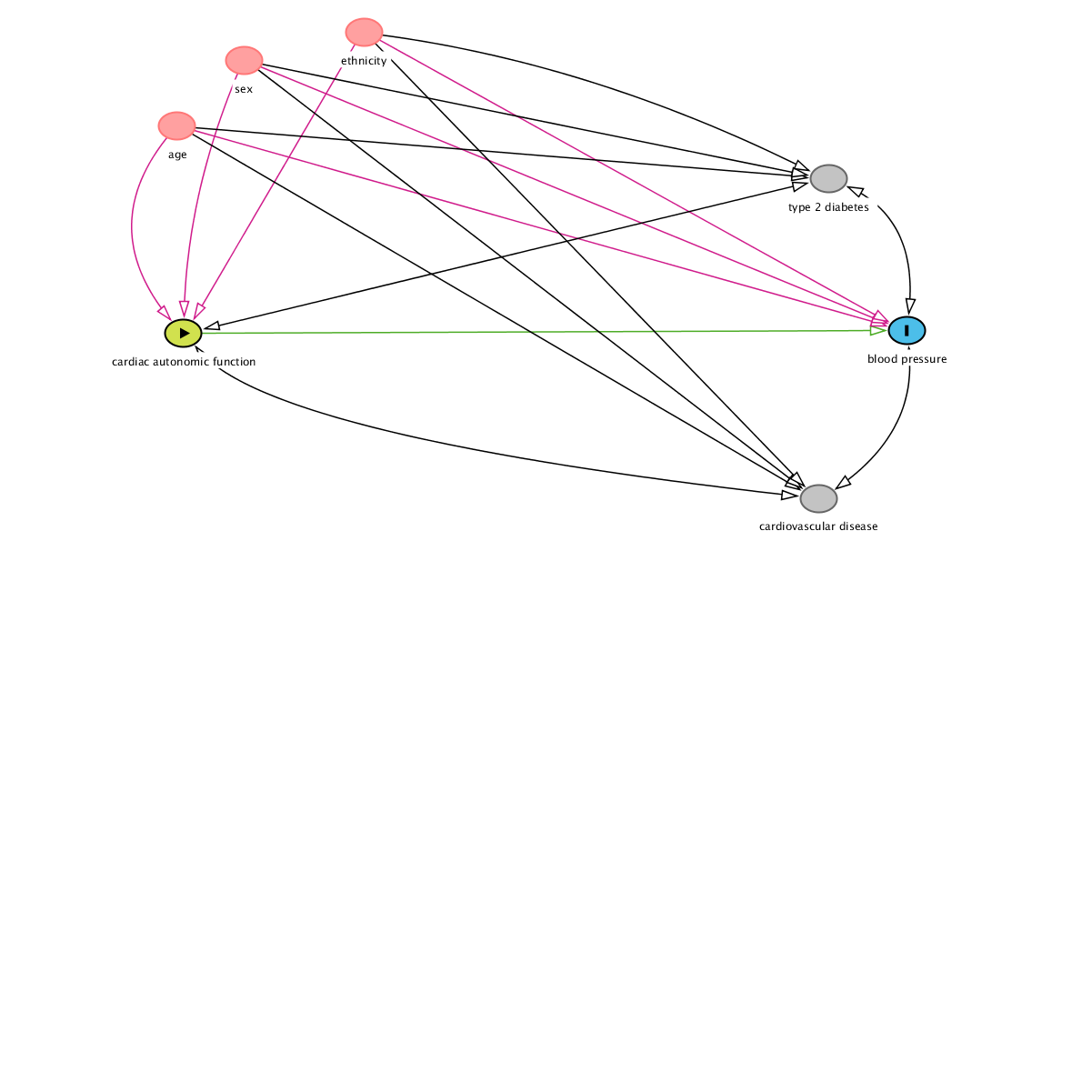


Directed acyclic graph implicating age, sex, and ethnicity, as potential confounders. In the case of type 2 diabetes and cardiovascular disease, there is directional ambiguity in the variable relationships. We therefore examined a separate model (Model 3) where we adjusted for type 2 diabetes and cardiovascular disease. Reassuringly, we obtained results that were not substantially different from those from Model 2.

Model 1: BP ~ HR(V) traits

Model 2: Model 1 + Age + Sex + Ethnicity

Model 3: Model 2 + CVD + T2DM

**Supplementary Figure S3. Assumptions and rationale of two-sample Mendelian randomization**


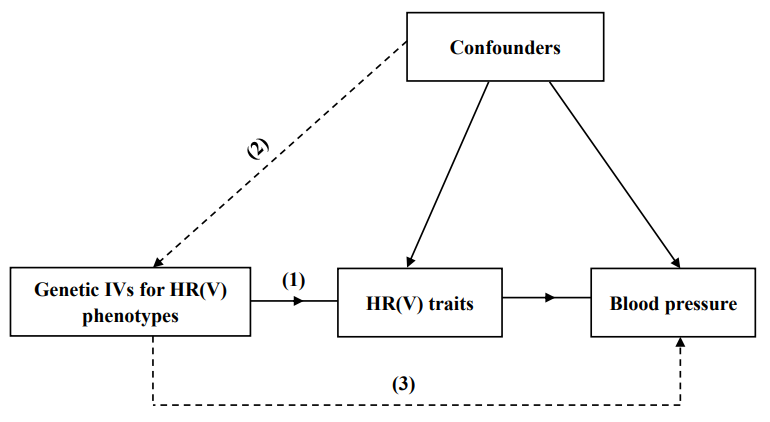


Abbreviations: IVs, instrumental variables; HR(V), heart rate (variability).

(1) the relevance assumption: there is a strong relation between IV and HR(V) trait

(2) the independence assumption: there is no relation between IV and any confounder in the HR(V)- blood pressure relationship

(3) the exclusion restriction assumption: the effect of the IV on blood pressure is mediated completely through the HR(V) trait.

**Supplementary Figure S4.** **Mendelian randomization scatterplots of HR(V) traits to blood pressure**


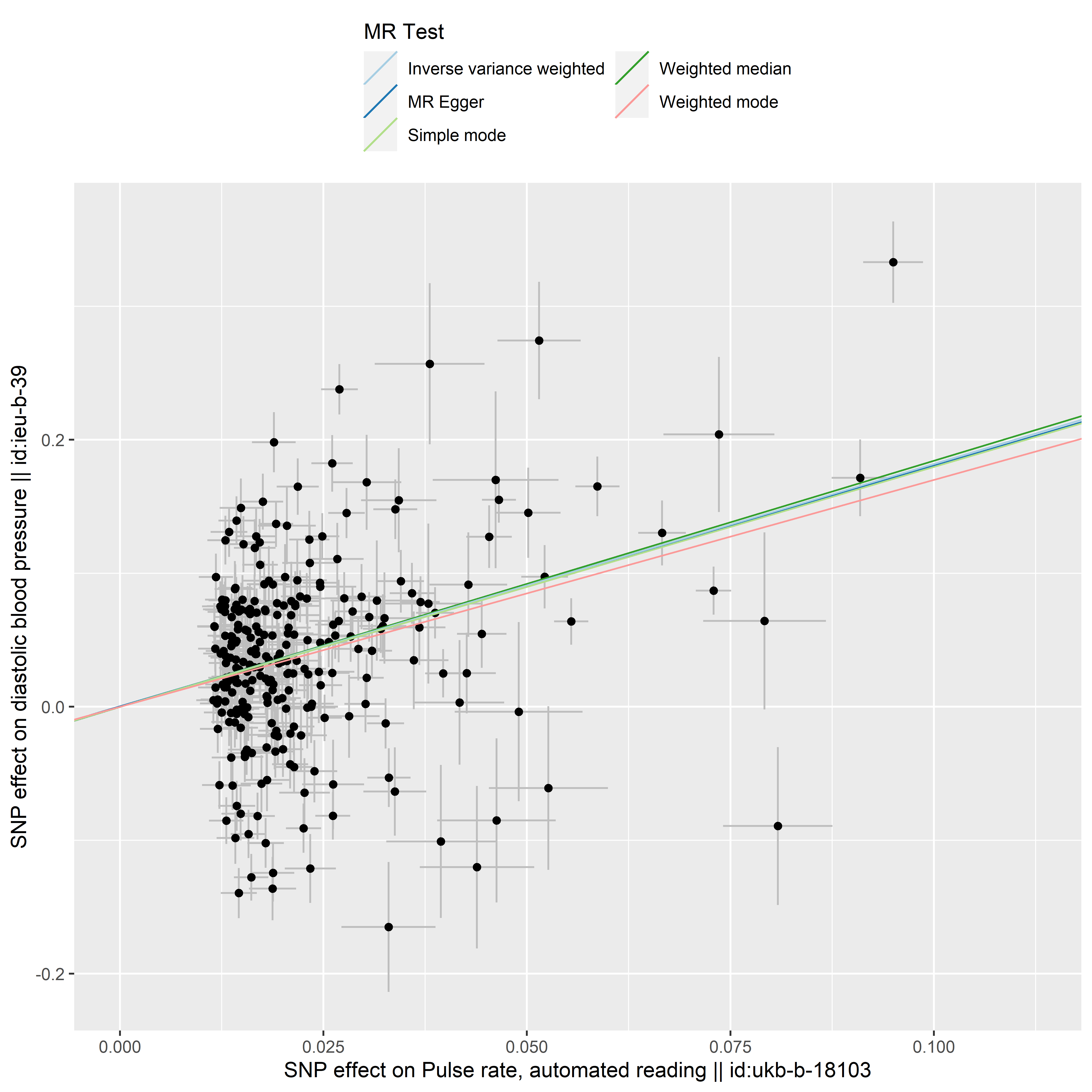

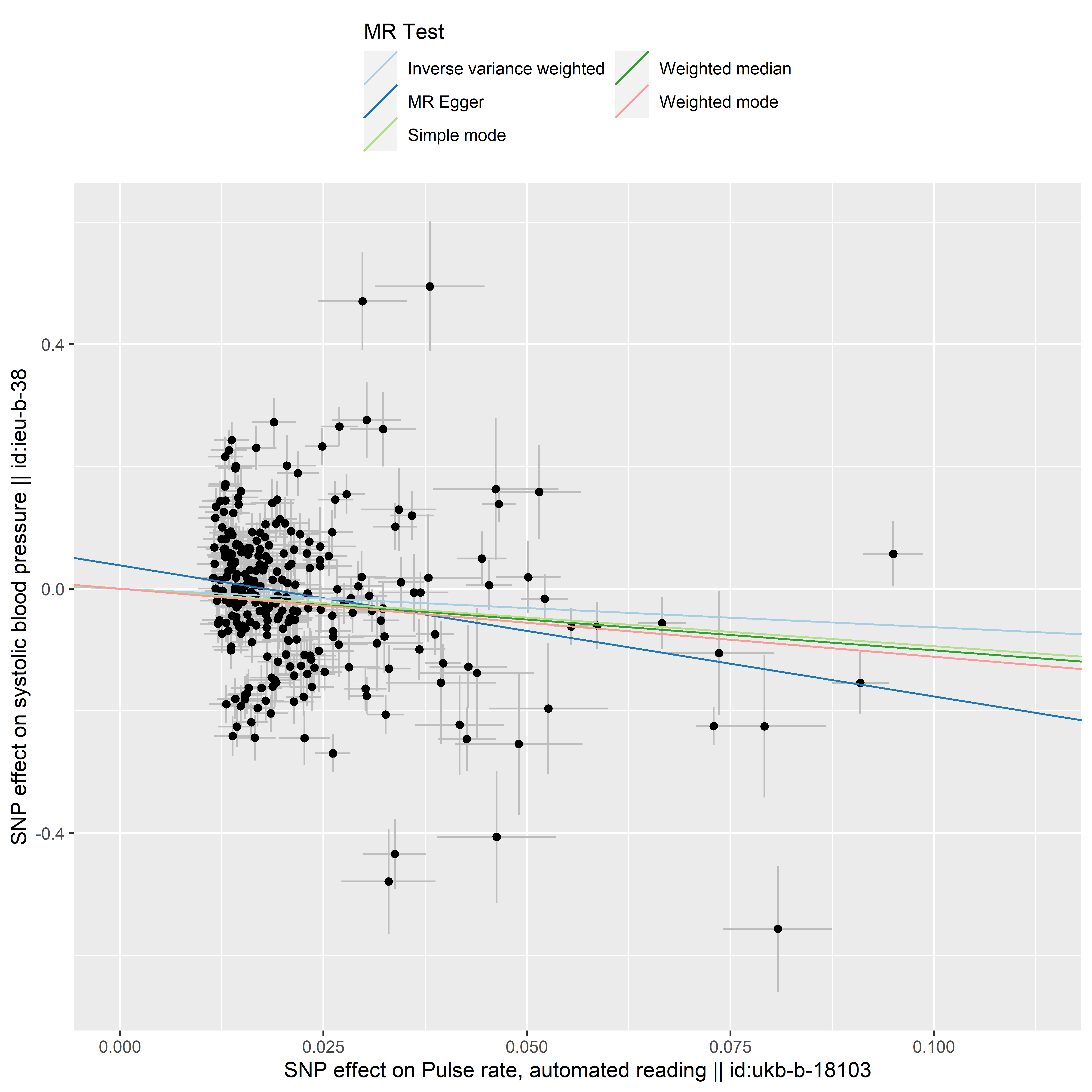


Resting HR (exposure) vs. SBP (outcome) Resting HR (exposure) vs. DBP (outcome)


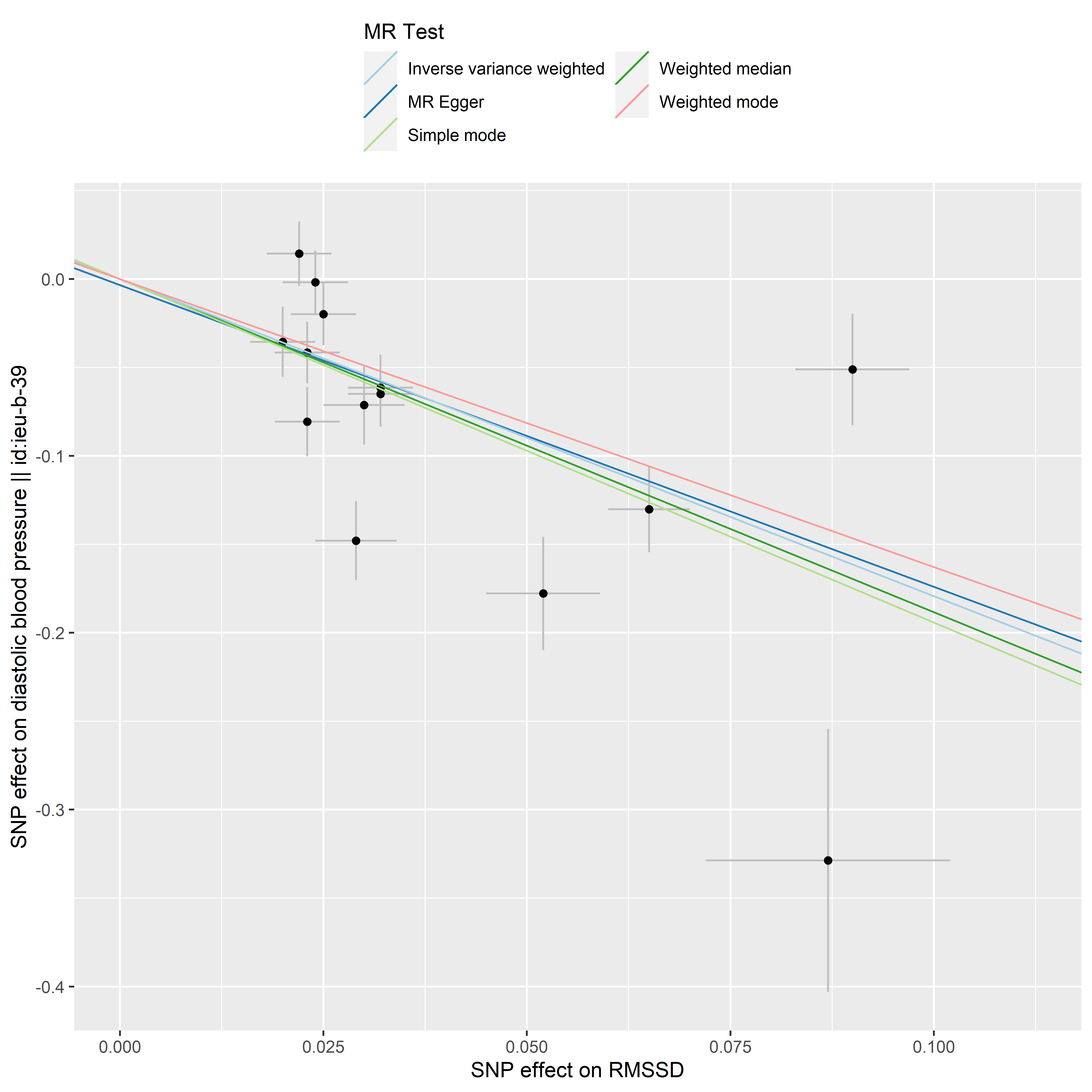

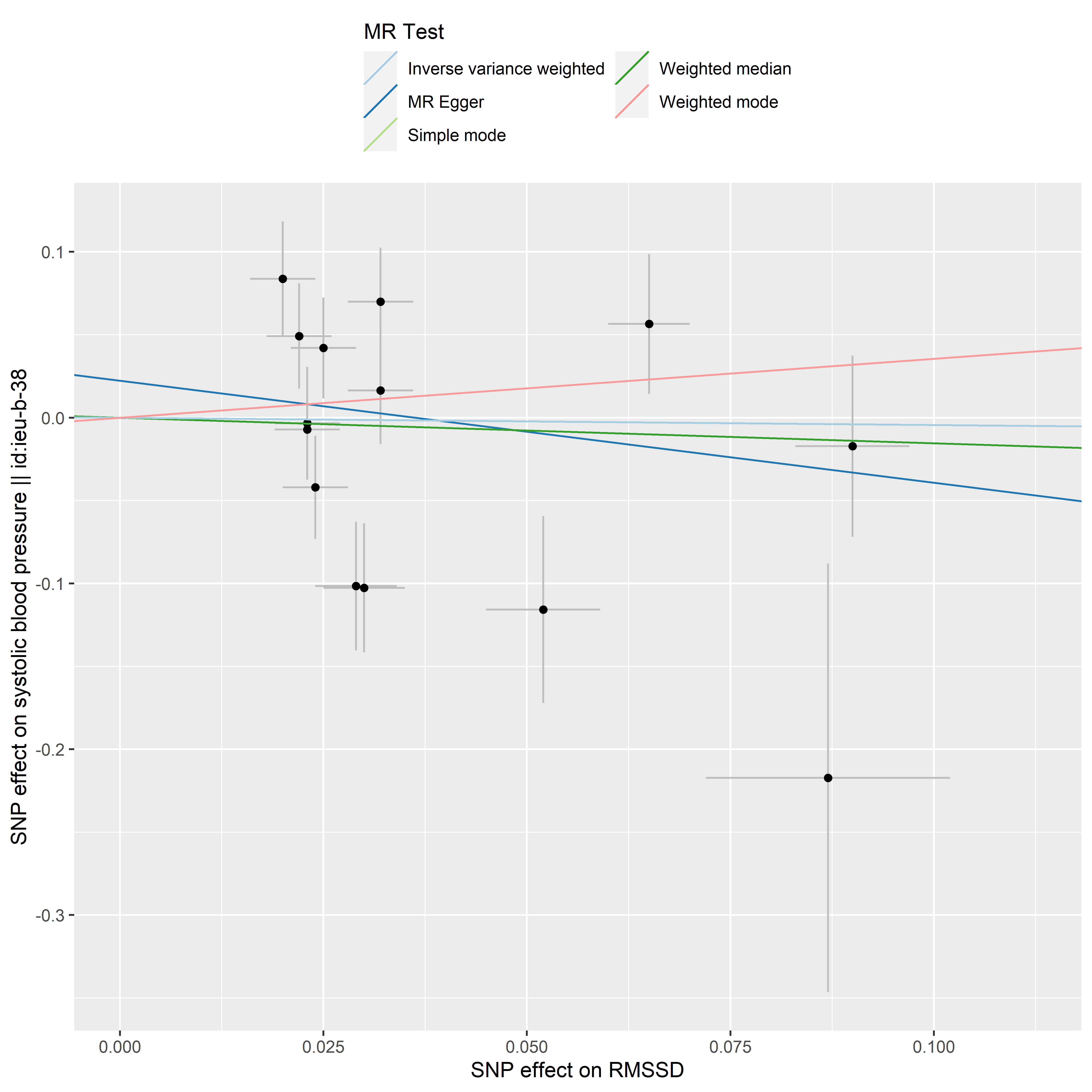


RMSSD (exposure) vs. SBP (outcome) RMSSD (exposure) vs. DBP (outcome)


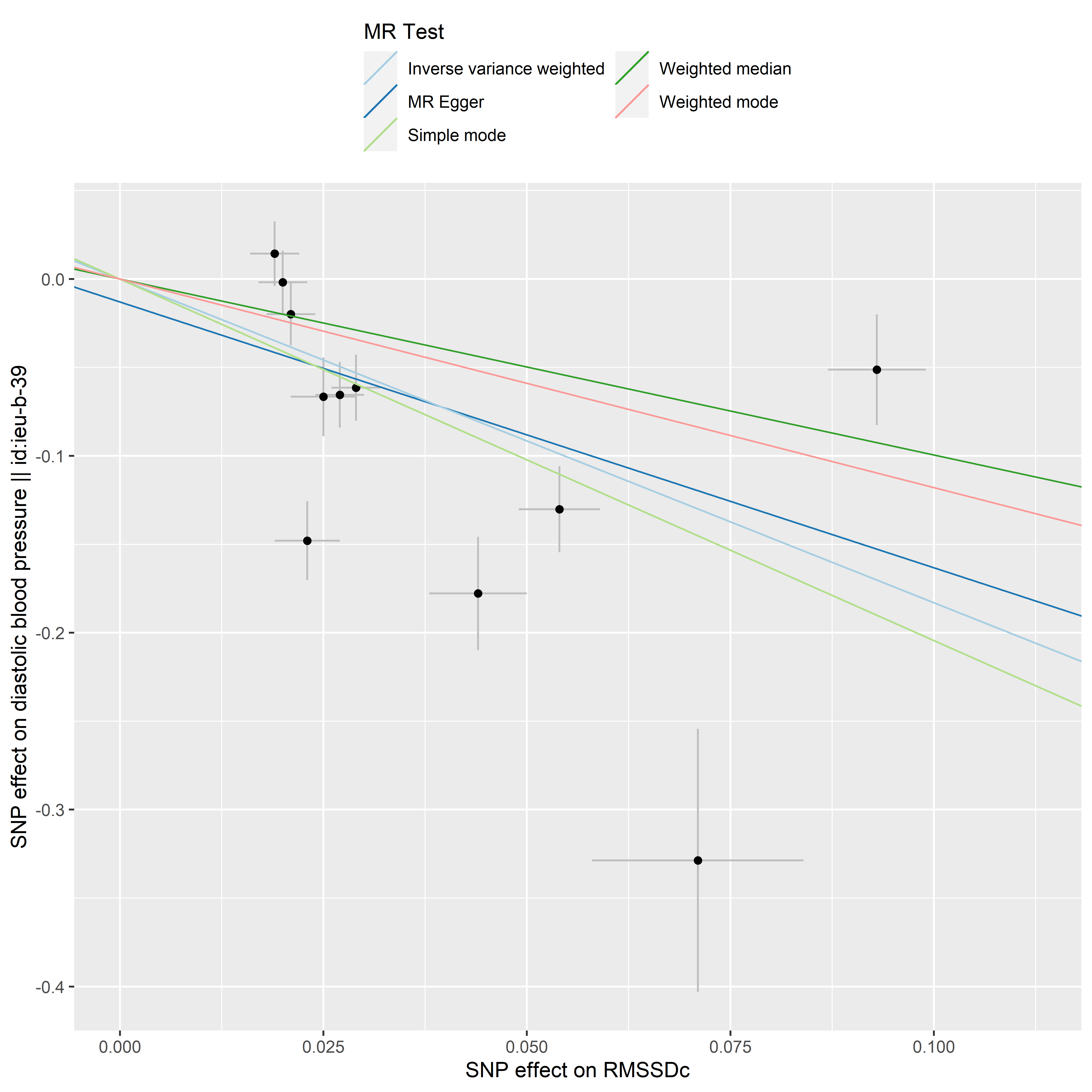

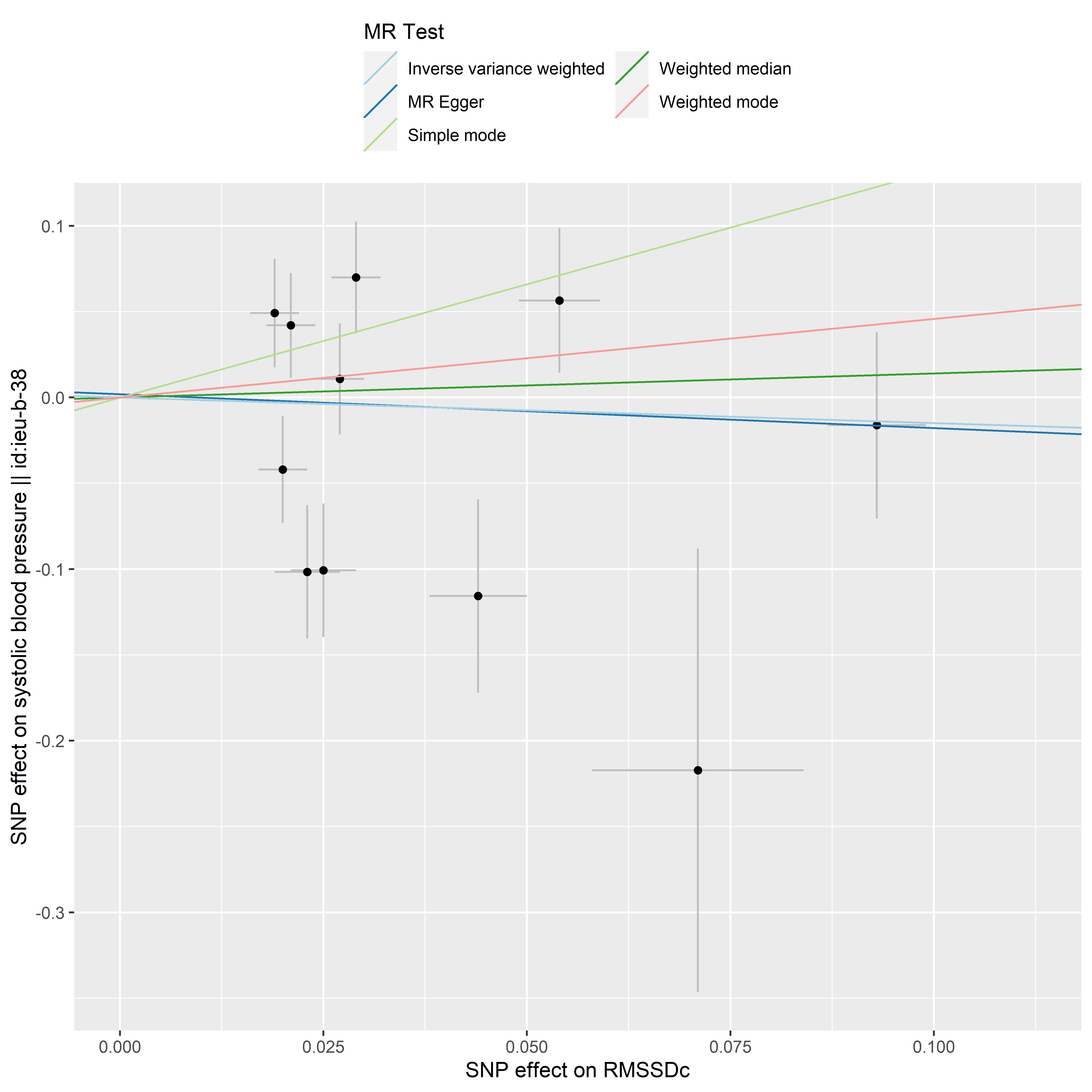


RMSSDc (exposure) vs. SBP (outcome) RMSSDc (exposure) vs. DBP (outcome)


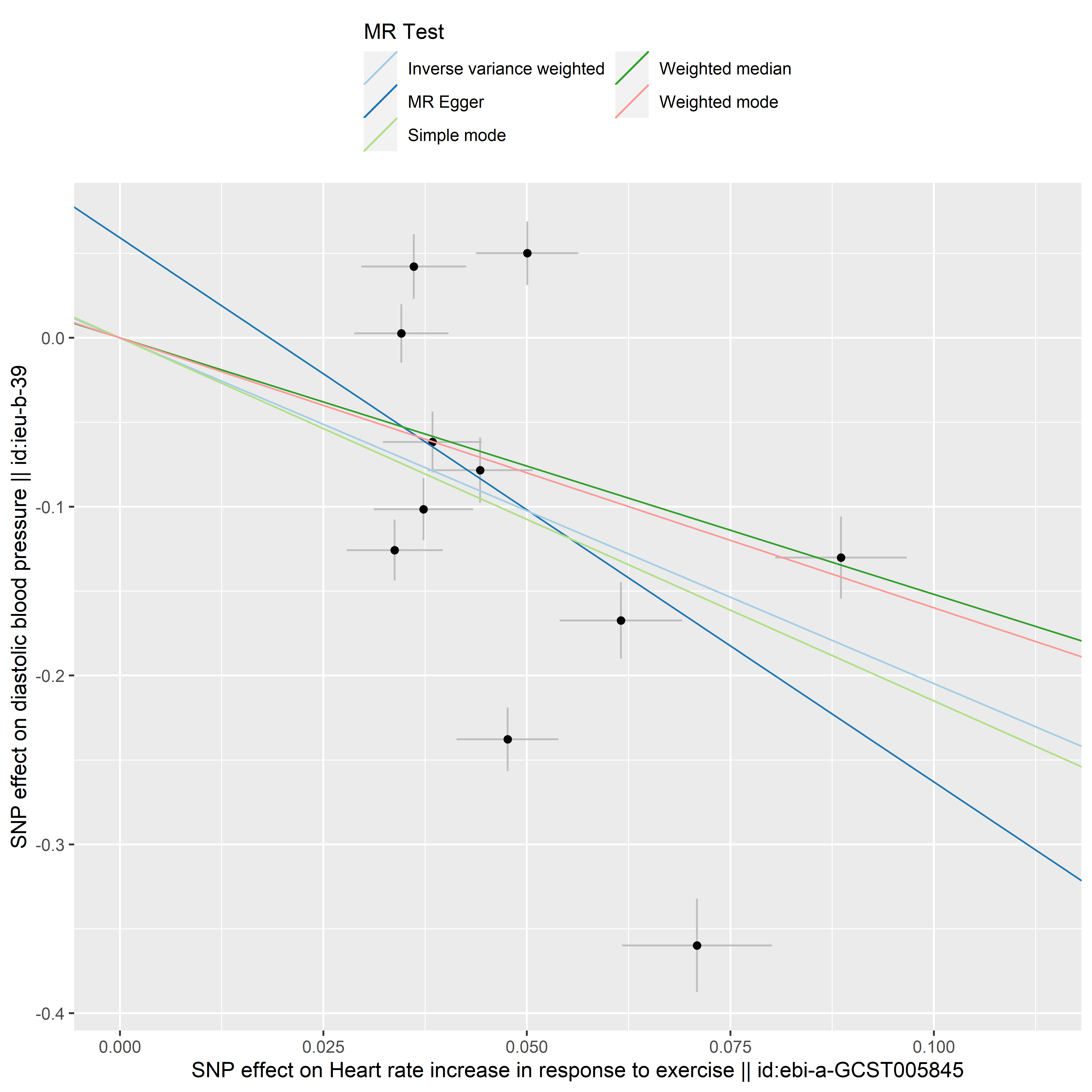

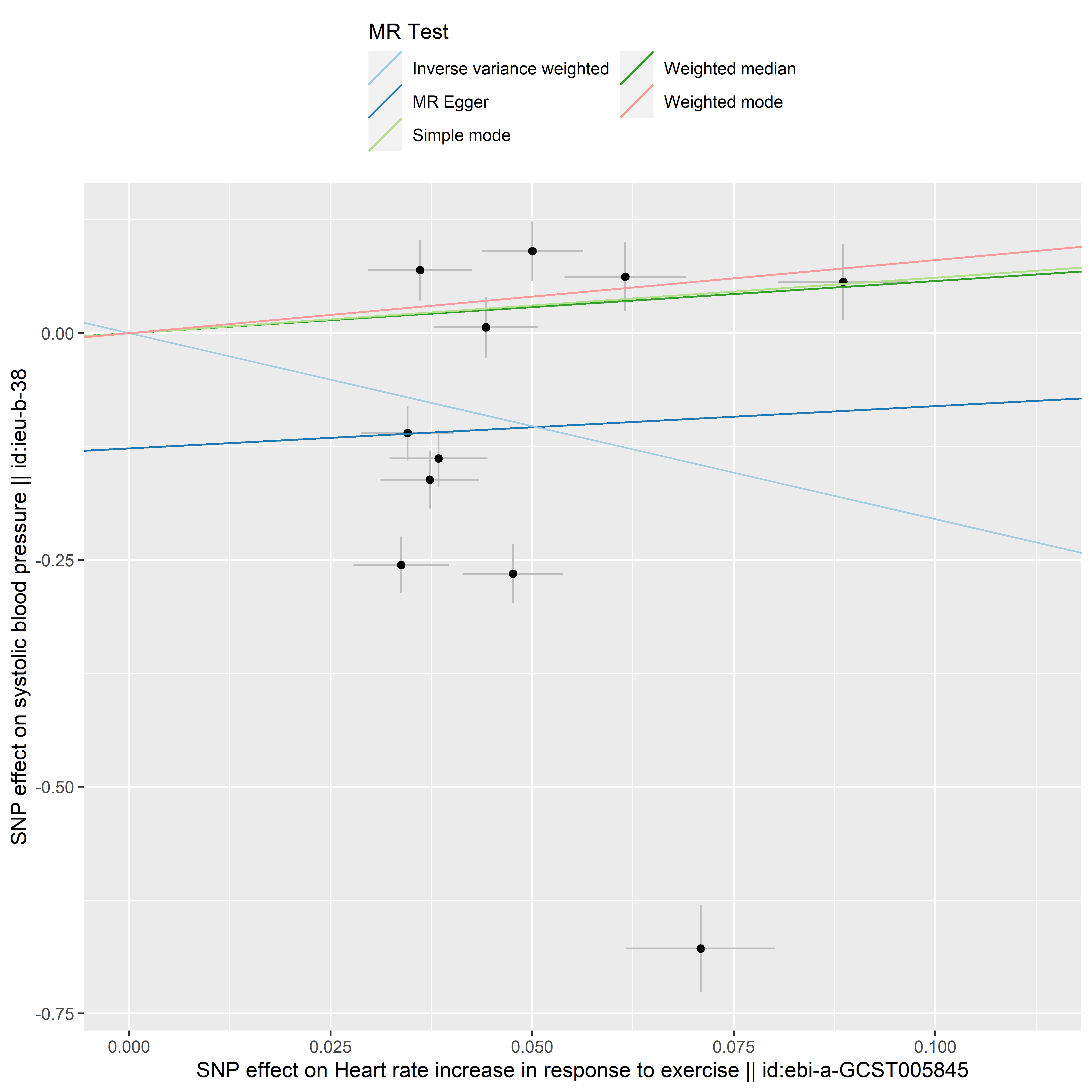


HR increase (exposure) vs. SBP (outcome) HR increase (exposure) vs. DBP (outcome)


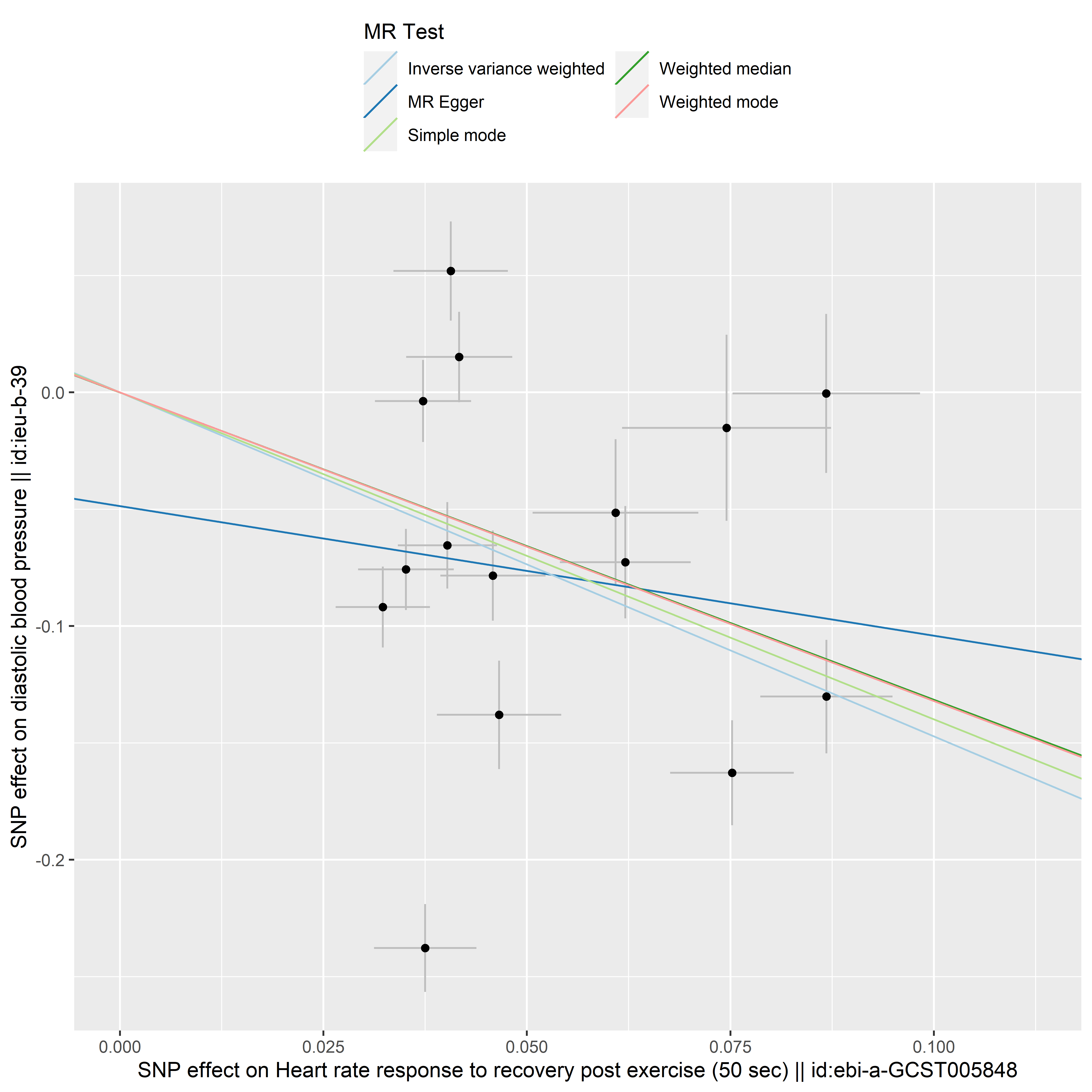

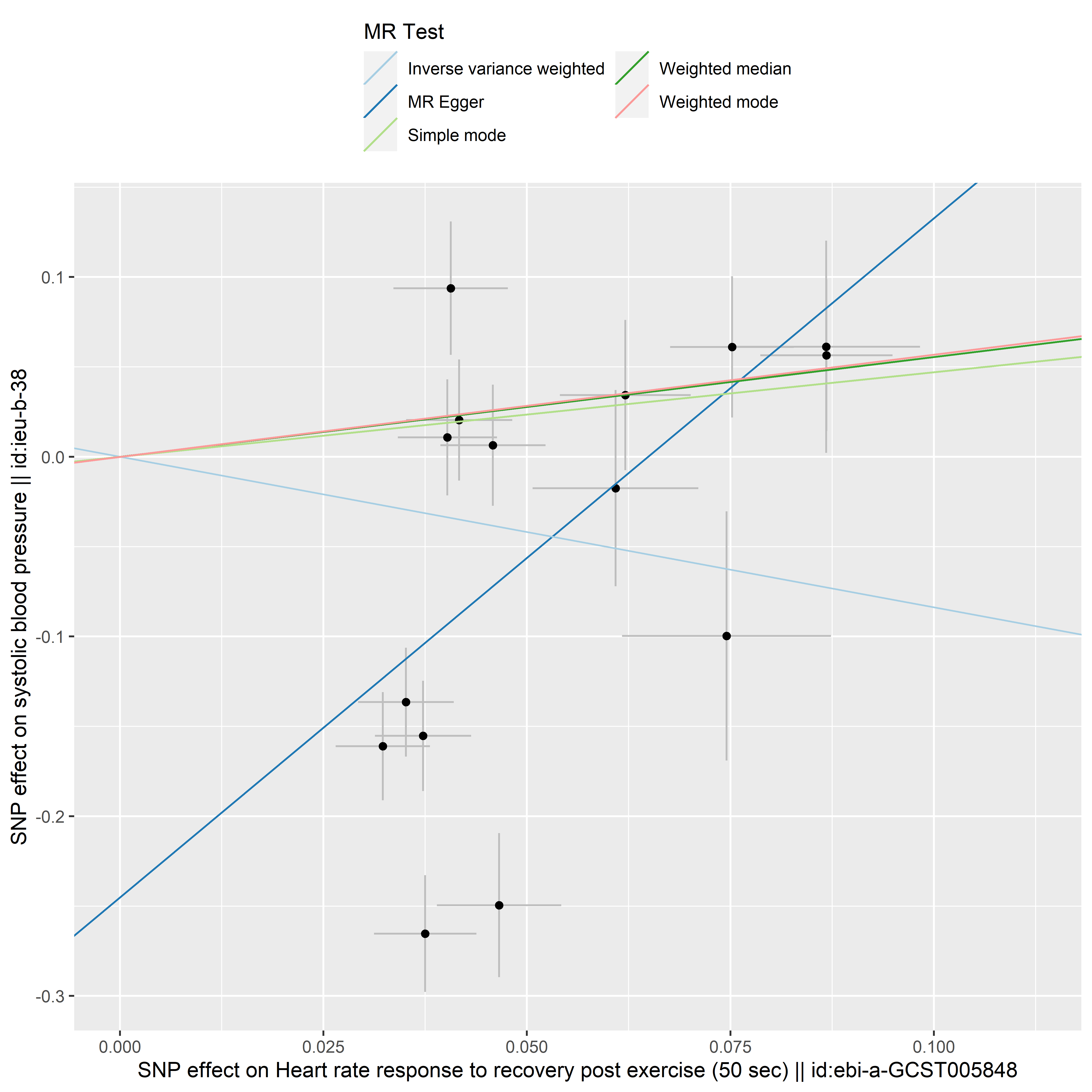


HR recovery_50s (exposure) vs. SBP (outcome) HR recovery_50s (exposure) vs. DBP (outcome)

Abbreviation: HR, heart rate; HR(V), heart rate (variability); RMSSD, root mean square of successive differences; RMSSDc, corrected root mean square of successive differences; SBP, systolic blood pressure; DBP, diastolic blood pressure.

**Supplementary Figure S5.** **Mendelian randomization single SNP forest plots and** **leave-one-out plots**


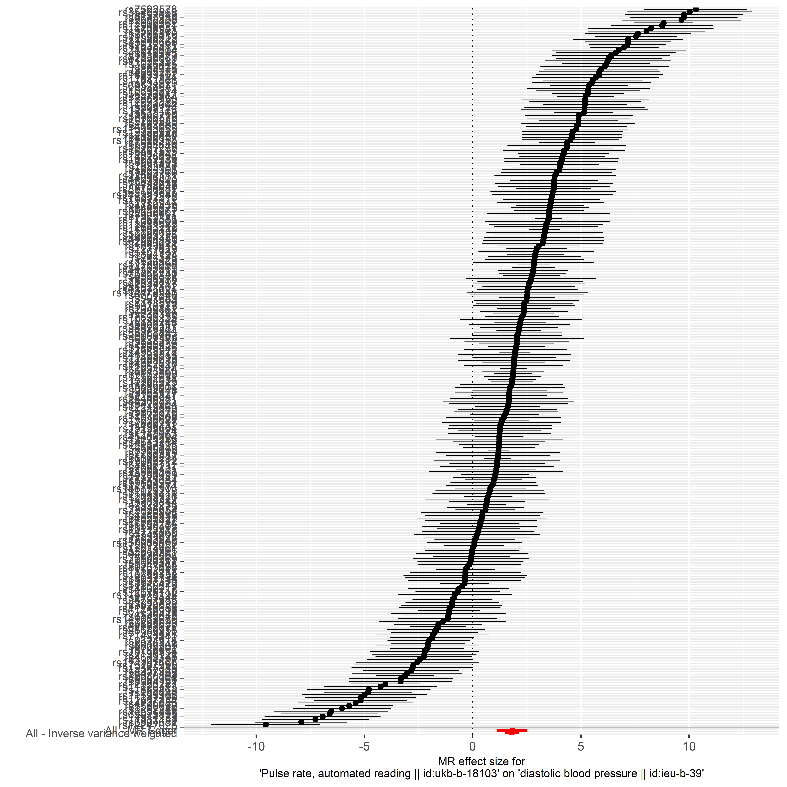

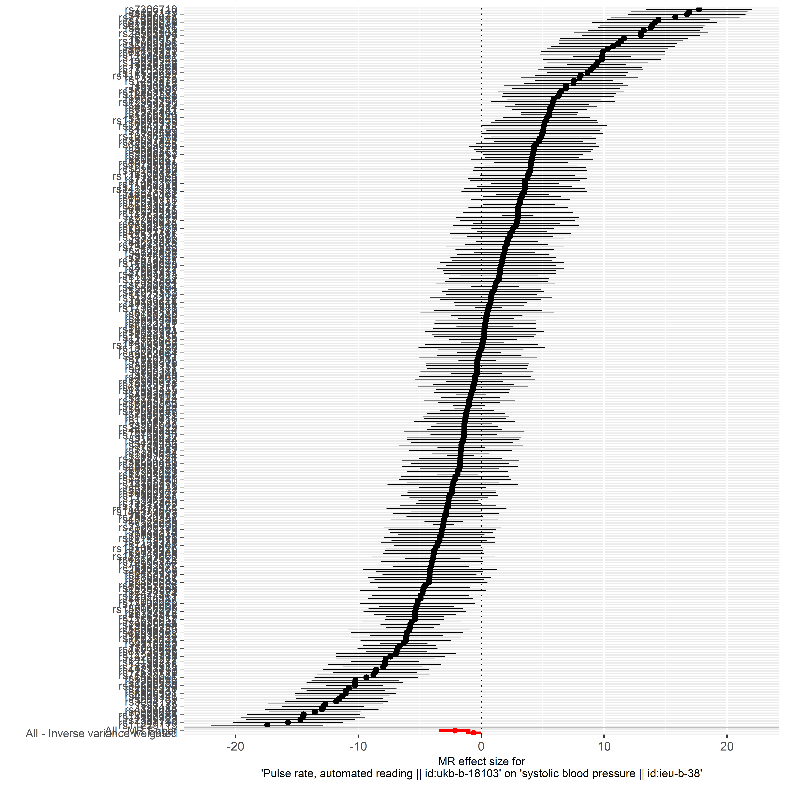


Single SNP forest plot**:** Resting HR vs. SBP Single SNP forest plot**:** Resting HR vs. DBP


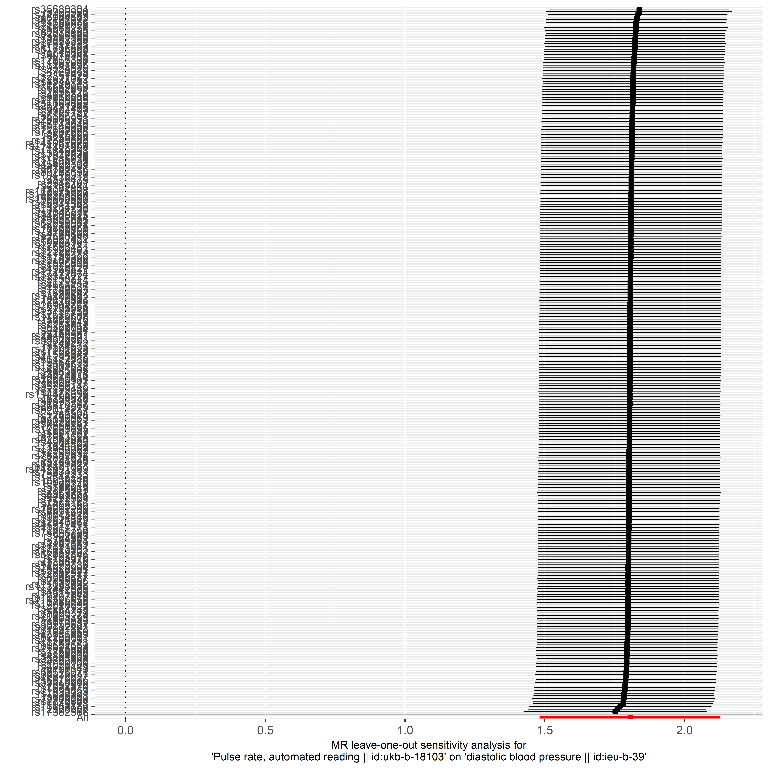

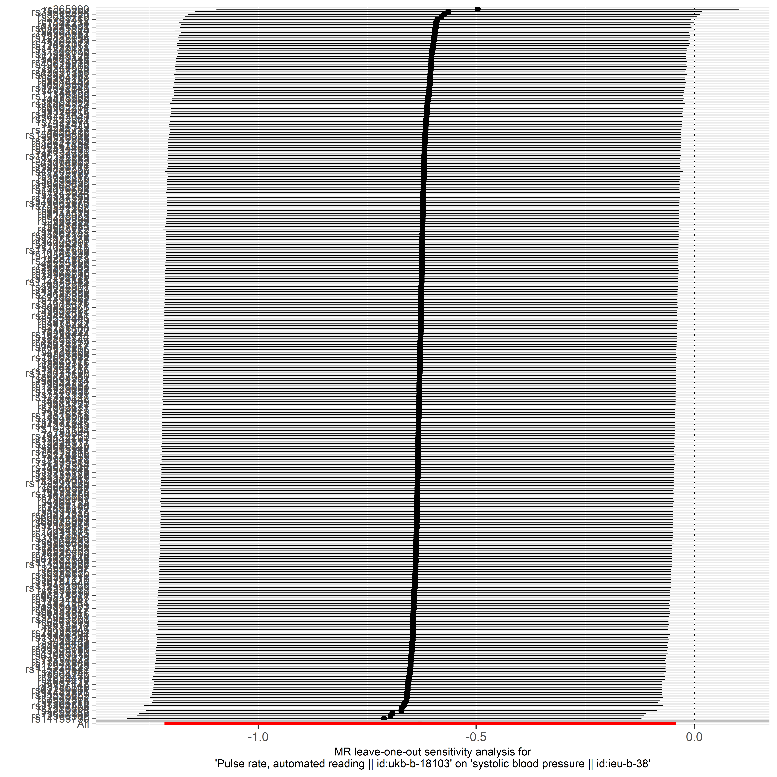


Leave-one-out plot**:** Resting HR vs. SBP Leave-one-out plot**:** Resting HR vs. DBP


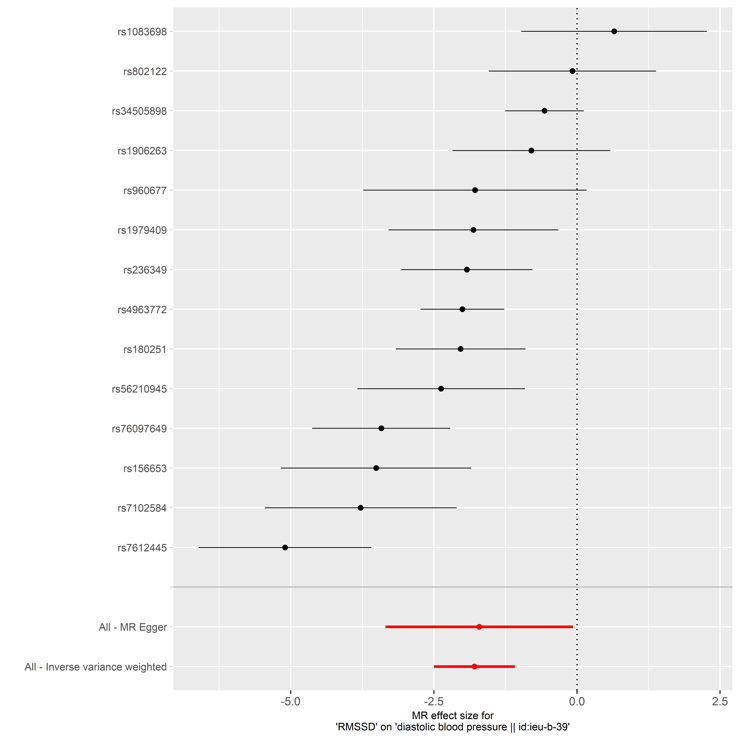

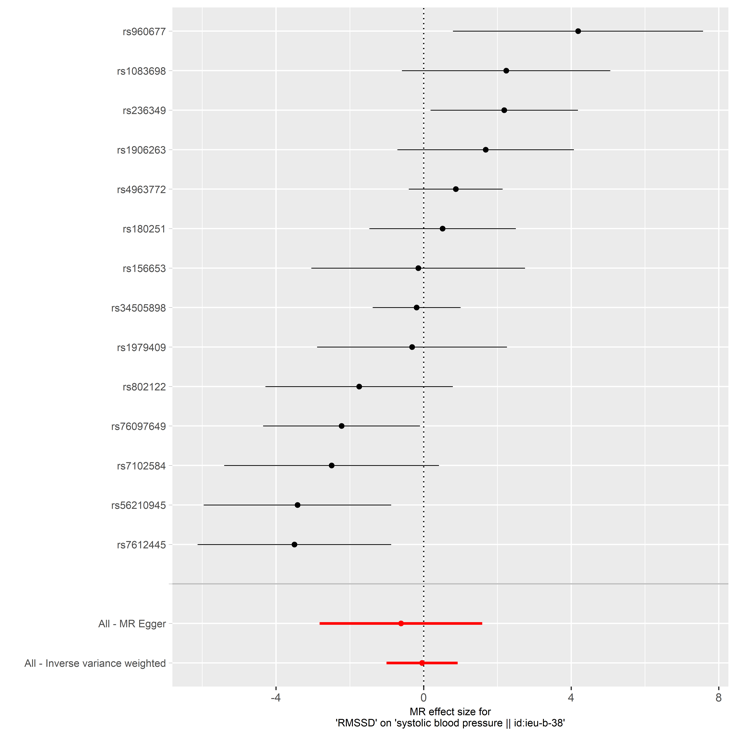


Single SNP forest plot**:** RMSSD vs. SBP Single SNP forest plot**:** RMSSD vs. DBP


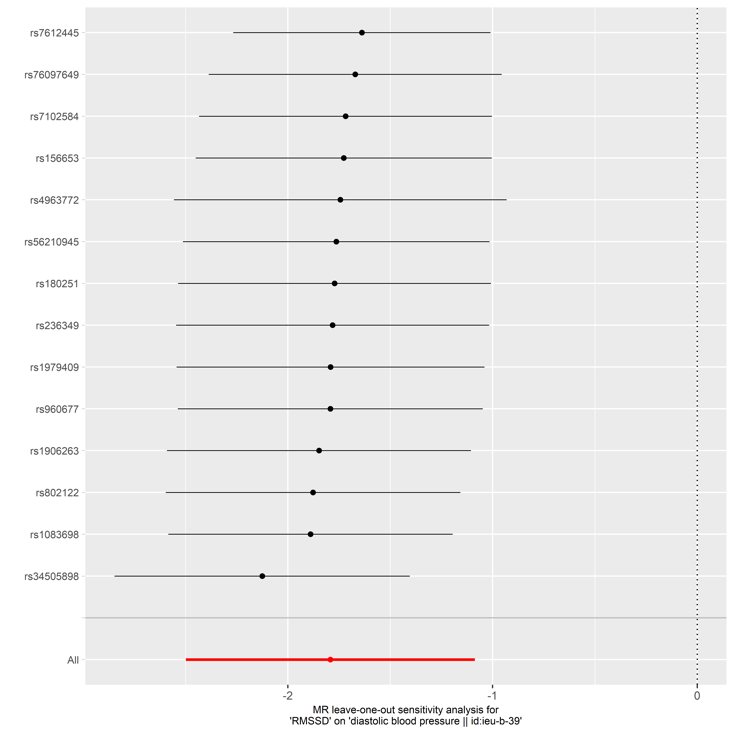

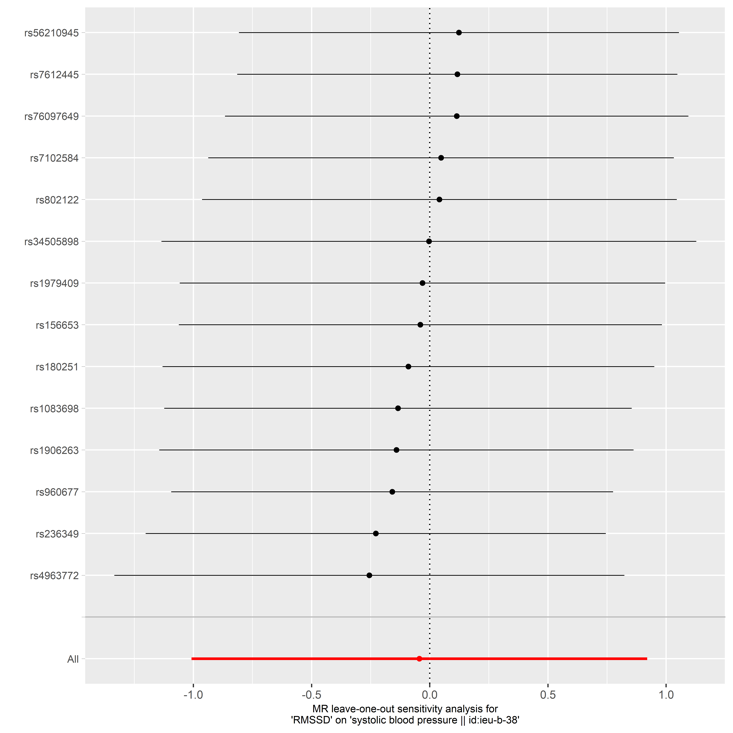


Leave-one-out plot**:** RMSSD vs. SBP Leave-one-out plot**:** RMSSD vs. DBP


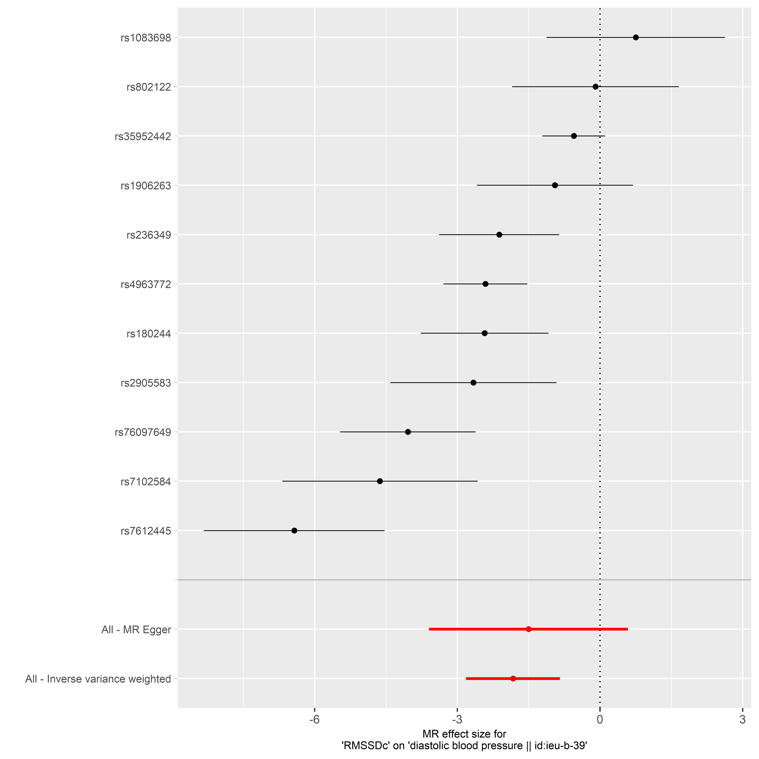

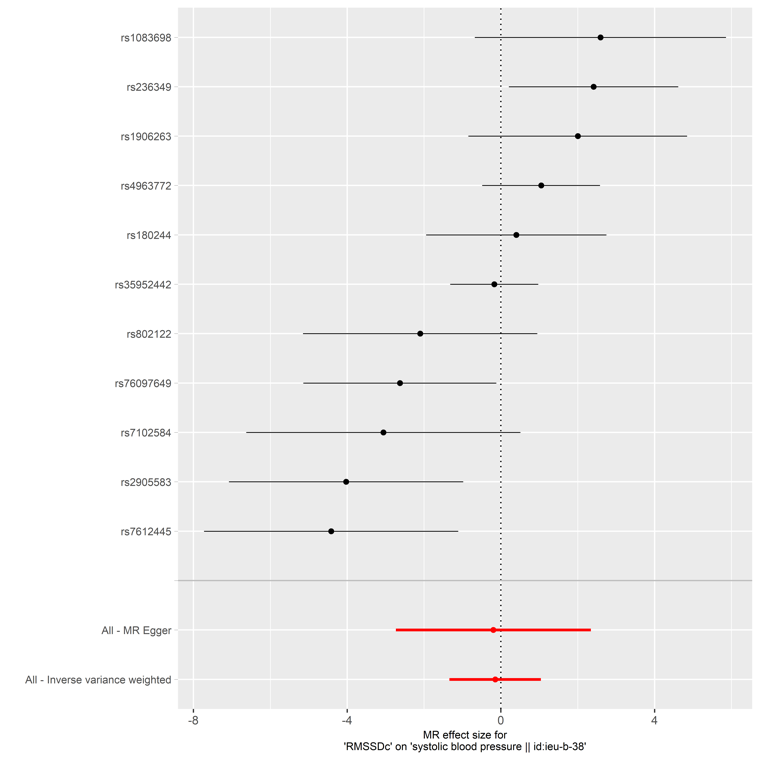


Single SNP forest plot**:** RMSSDc vs. SBP Single SNP forest plot**:** RMSSDc vs. DBP


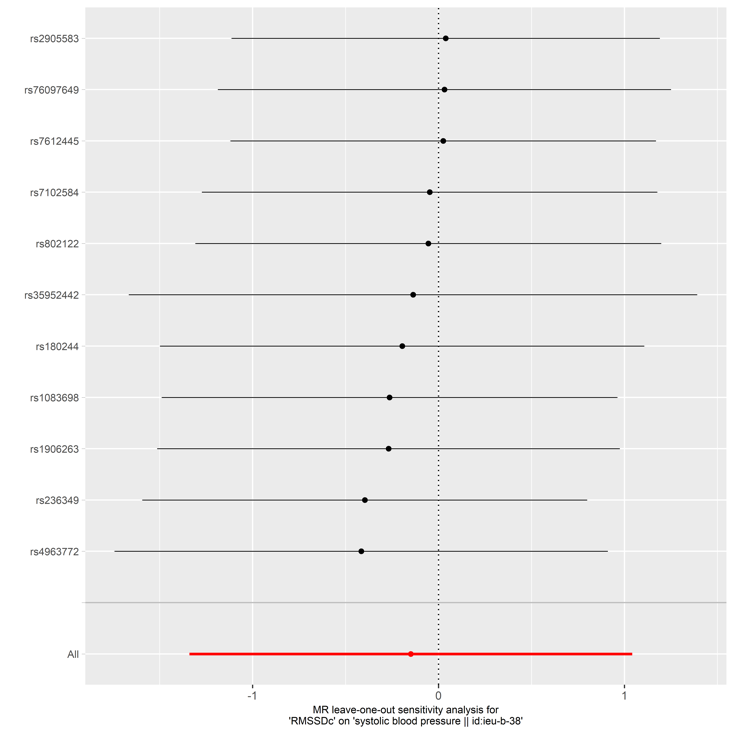

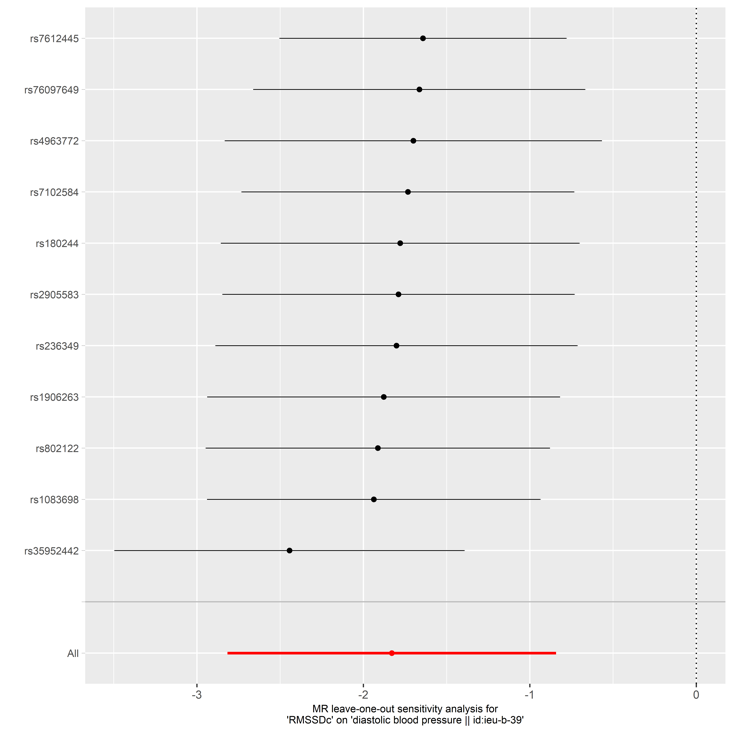


Leave-one-out plot**:** RMSSDc vs. SBP Leave-one-out plot**:** RMSSDc vs. DBP


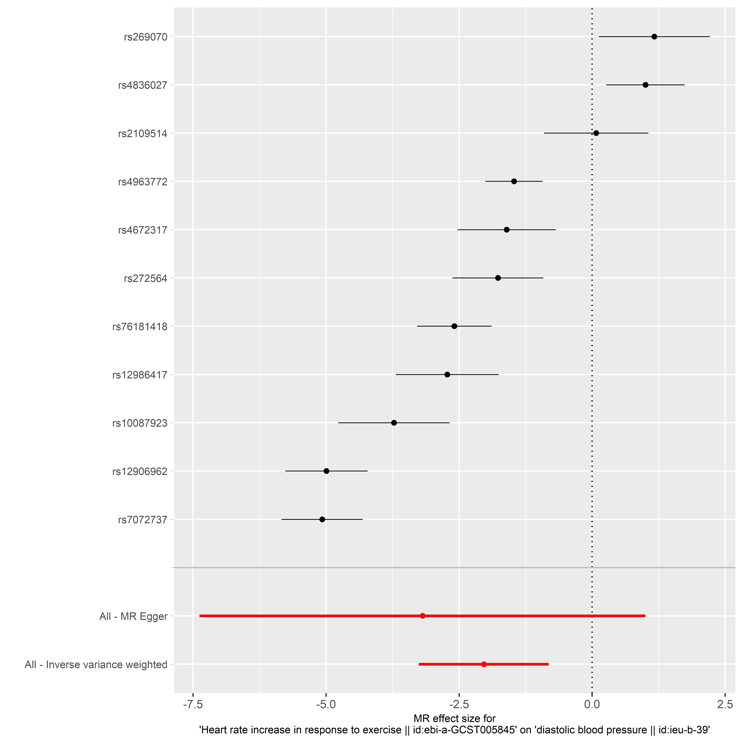

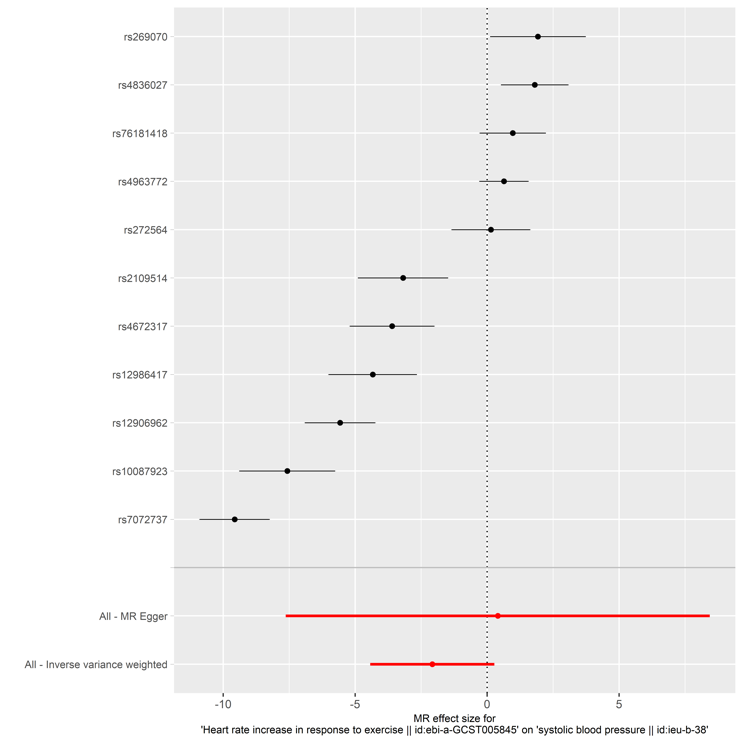


Single SNP forest plot**:** HR increase vs. SBP Single SNP forest plot**:** HR increase vs. DBP


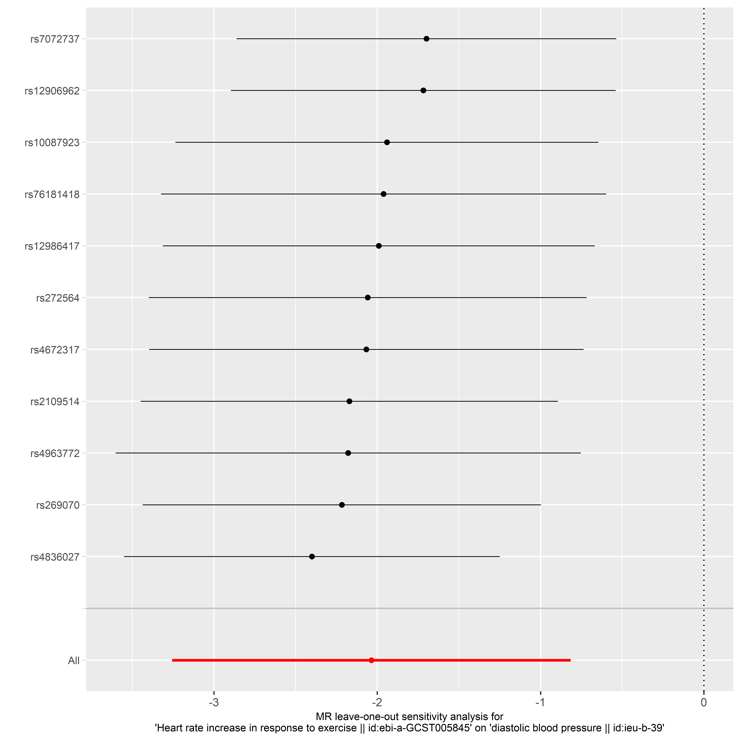

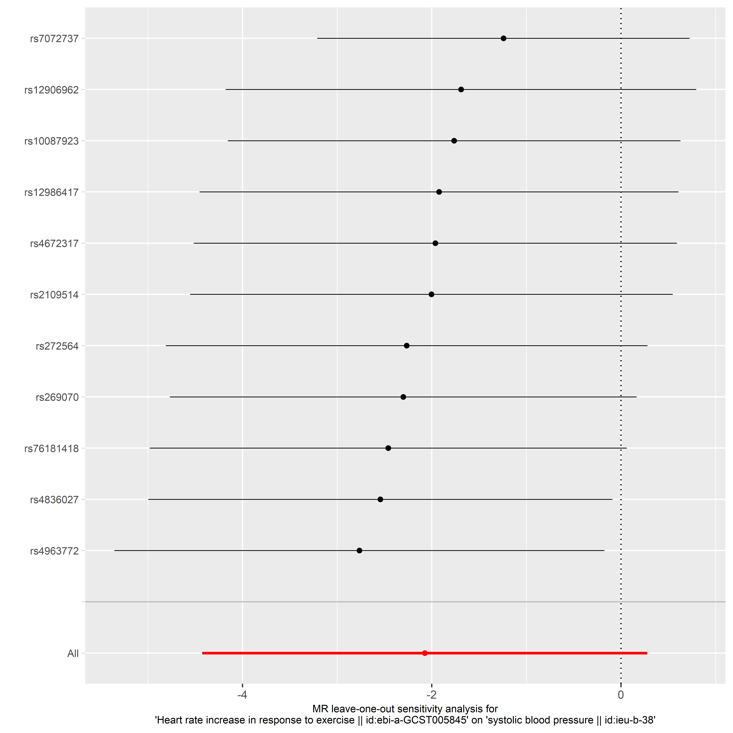


Leave-one-out plot**:** HR increase vs. SBP Leave-one-out plot**:** HR increase vs. DBP


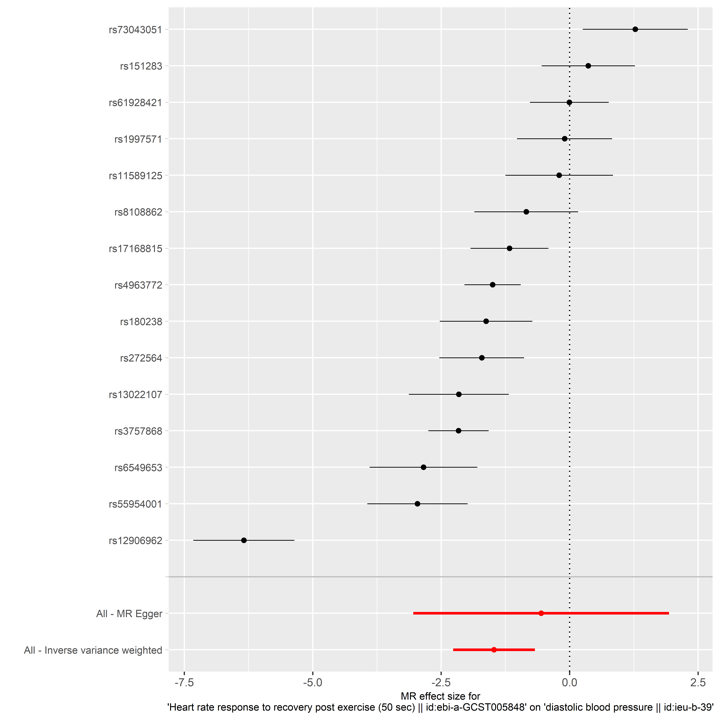

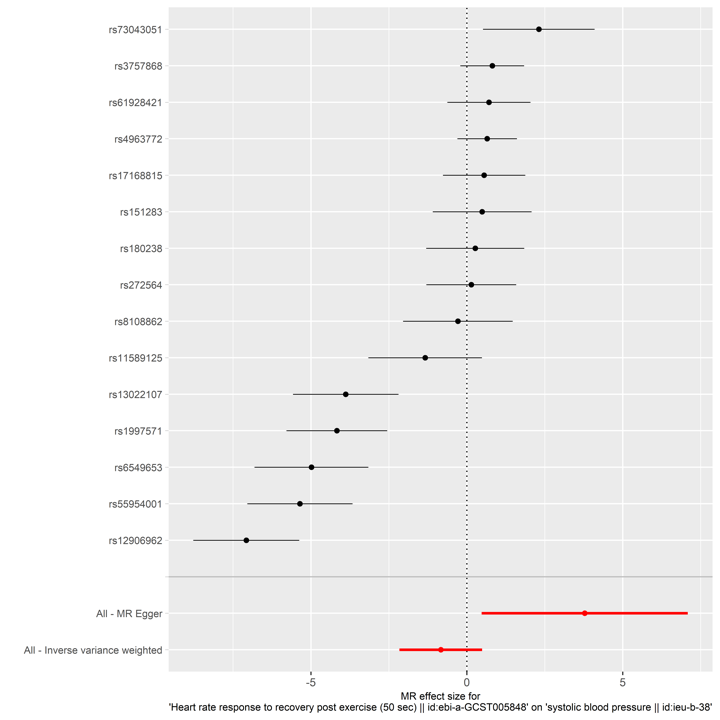


Single SNP forest plot**:** HR recovery_50s vs. SBP HR recovery_50s vs. DBP


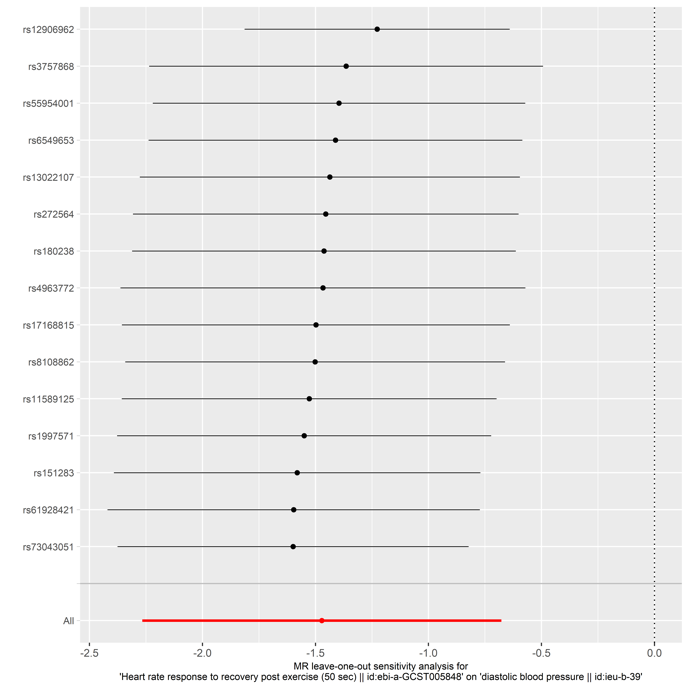

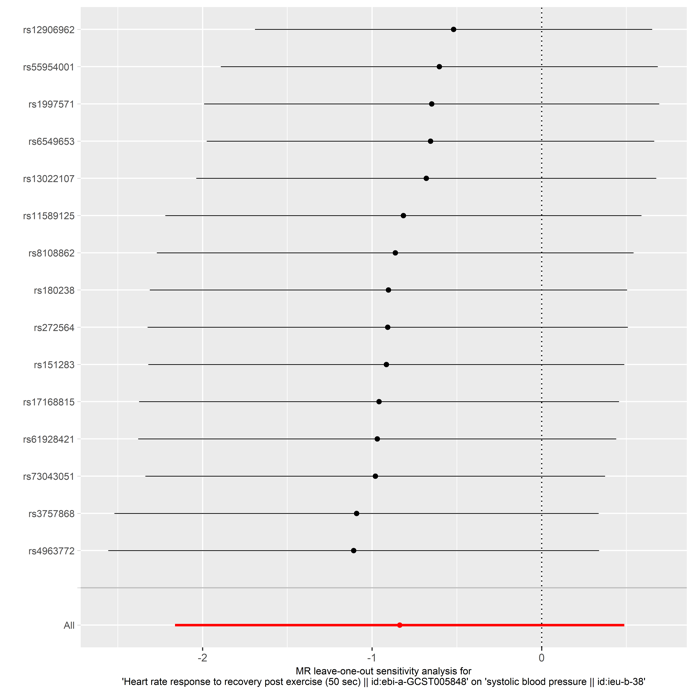


Leave-one-out plot**:** HR recovery_50s vs. SBP HR recovery_50s vs. DBP

Abbreviation: HR, heart rate; HR(V), heart rate (variability); RMSSD, root mean square of successive differences; RMSSDc, corrected root mean square of successive differences; SBP, systolic blood pressure; DBP, diastolic blood pressure.

**Supplementary Document 1. The list of all ICBP authors from the 77 ICBP studies contributing to the ICBP meta-analysis**

Authors & Contributors from the International Consortium of Blood Pressure (ICBP) associated with the 2018 Nature Genetics publication: “Genetic analysis of over one million people identifies 535 new loci associated with blood pressure traits.”

Evangelos Evangelou^1,2^, Helen R Warren^3,4^, He Gao^1,5^, Georgios Ntritsos^2^, Niki Dimou^2^, Tonu Esko^16,17^, Reedik Mägi^16^, Lili Milani^16^, Peter Almgren^18^, Thibaud Boutin^19^, Stéphanie Debette^20,21^, Jun Ding^22^, Franco Giulianini^23^, Elizabeth G Holliday^24^, Anne U Jackson^25^, Ruifang Li-Gao^26^, Wei-Yu Lin^27^, Jian'an Luan^28^, Massimo Mangino^29,30^, Christopher Oldmeadow^24^, Bram Peter Prins^31^, Yong Qian^22^, Muralidharan Sargurupremraj^21^, Nabi Shah^32,33^, Praveen Surendran^27^, Sébastien Thériault^34,35^, Niek Verweij^17,36,37^, Sara M Willems^28^, Jing-Hua Zhao^28^, Philippe Amouyel^38^, John Connell^39^, Renée de Mutsert^26^, Alex SF Doney^32^, Martin Farrall^40,41^, Cristina Menni^29^, Andrew D Morris^42^, Raymond Noordam^43^, Guillaume Paré^34^, Neil R Poulter^44^, Denis C Shields^45^, Alice Stanton^46^, Simon Thom^47^, Gonçalo Abecasis^48^, Najaf Amin^49^, Dan E Arking^50^, Kristin L Ayers^51,52^, Caterina M Barbieri^53^, Chiara Batini^54^, Joshua C Bis^55^, Tineka Blake^54^, Murielle Bochud^56^, Michael Boehnke^25^, Eric Boerwinkle^57^, Dorret I Boomsma^58^, Erwin P Bottinger^59^, Peter S Braund^60,61^, Marco Brumat^62^, Archie Campbell^63,64^, Harry Campbell^65^, Aravinda Chakravarti^50^, John C Chambers^1,5,66-68^, Ganesh Chauhan^69^, Marina Ciullo^70,71^, Massimiliano Cocca^72^, Francis Collins^73^, Heather J Cordell^51^, Gail Davies^74,75^, Martin H de Borst^76^, Eco J de Geus^58^, Ian J Deary^74,75^, Joris Deelen^77^, Fabiola Del Greco M^78^, Cumhur Yusuf Demirkale^79^, Marcus Dörr^80,81^, Georg B Ehret^50,82^, Roberto Elosua^83,84^, Stefan Enroth^85^, A Mesut Erzurumluoglu^54^, Teresa Ferreira^86,87^, Mattias Frånberg^88-90^, Oscar H Franco^91^, Ilaria Gandin^62^, Paolo Gasparini^62,72^, Vilmantas Giedraitis^92^, Christian Gieger^93-95^, Giorgia Girotto^62,72^, Anuj Goel^40,41^, Alan J Gow^74,96^, Vilmundur Gudnason^97,98^, Xiuqing Guo^99^, Ulf Gyllensten^85^, Anders Hamsten^88,89^, Tamara B Harris^100^, Sarah E Harris^63,74^, Catharina A Hartman^101^, Aki S Havulinna^102,103^, Andrew A Hicks^78^, Edith Hofer^104,105^, Albert Hofman^91,106^, Jouke-Jan Hottenga^58^, Jennifer E Huffman^19,107,108^, Shih-Jen Hwang^107,108^, Erik Ingelsson^109,110^, Alan James^111,112^, Rick Jansen^113^, Marjo-Riitta Jarvelin^1,5,114-116^, Roby Joehanes^107,117^, Åsa Johansson^85^, Andrew D Johnson^107,118^, Peter K Joshi^65^, Pekka Jousilahti^102^, J Wouter Jukema^119^, Antti Jula^102^, Mika Kähönen^120,121^, Sekar Kathiresan^17,36,122^, Bernard D Keavney^123,124^, Kay-Tee Khaw^125^, Paul Knekt^102^, Joanne Knight^126^, Ivana Kolcic^127^, Jaspal S Kooner^5,67,68,128^, Seppo Koskinen^102^, Kati Kristiansson^102^, Zoltan Kutalik^56,129^, Maris Laan^130^, Marty Larson^107^, Lenore J Launer^100^, Benjamin Lehne^1^, Terho Lehtimäki^131,132^, David CM Liewald^74,75^, Li Lin^82^, Lars Lind^133^, Cecilia M Lindgren^40,87,134^, YongMei Liu^135^, Ruth JF Loos^28,59,136^, Lorna M Lopez^74,137,138^, Yingchang Lu^59^, Leo-Pekka Lyytikäinen^131,132^, Anubha Mahajan^40^, Chrysovalanto Mamasoula^139^, Jaume Marrugat^83^, Jonathan Marten^19^, Yuri Milaneschi^140^, Anna Morgan^62^, Andrew P Morris^40,141^, Alanna C Morrison^142^, Peter J Munson^79^, Mike A Nalls^143,144^, Priyanka Nandakumar^50^, Christopher P Nelson^60,61^, Teemu Niiranen^102,145^, Ilja M Nolte^146^, Teresa Nutile^70^, Albertine J Oldehinkel^147^, Ben A Oostra^49^, Paul F O'Reilly^148^, Elin Org^16^, Sandosh Padmanabhan^64,149^, Walter Palmas^150^, Aarno Palotie^103,151,152^, Alison Pattie^75^, Brenda WJH Penninx^140^, Markus Perola^102,103,153^, Annette Peters^94,95,154^, Ozren Polasek^127,155^, Peter P Pramstaller^78,156,157^, Quang Tri Nguyen^79^, Olli T Raitakari^158,159^, Rainer Rettig^161^, Kenneth Rice^162^, Paul M Ridker^23,163^, Janina S Ried^94^, Harriëtte Riese^147^, Samuli Ripatti^103,164^, Antonietta Robino^72^, Lynda M Rose^23^, Jerome I Rotter^99^, Igor Rudan^165^, Daniela Ruggiero^70,71^, Yasaman Saba^166^, Cinzia F Sala^53^, Veikko Salomaa^102^, Nilesh J Samani^60,61^, Antti-Pekka Sarin^103^, Reinhold Schmidt^104^, Helena Schmidt^166^, Nick Shrine^54^, David Siscovick^167^, Albert V Smith^97,98^, Harold Snieder^146^, Siim Sõber^130^, Rossella Sorice^70^, John M Starr^74,168^, David J Stott^169^, David P Strachan^170^, Rona J Strawbridge^88,89^, Johan Sundström^133^, Morris A Swertz^171^, Kent D Taylor^99^, Alexander Teumer^81,172^, Martin D Tobin^54^, Maciej Tomaszewski^123,124^, Daniela Toniolo^53^, Michela Traglia^53^, Stella Trompet^119,173^, Jaakko Tuomilehto^174-177^, Christophe Tzourio^21^, André G Uitterlinden^91,178^, Ahmad Vaez^146,179^, Peter J van der Most^146^, Cornelia M van Duijn^49^, Germaine C Verwoert^91^, Veronique Vitart^19^, Uwe Völker^81,180^, Peter Vollenweider^181^, Dragana Vuckovic^62,182^, Hugh Watkins^40,41^, Sarah H Wild^183^, Gonneke Willemsen^58^, James F Wilson^19,65^, Alan F Wright^19^, Jie Yao^99^, Tatijana Zemunik^184^, Weihua Zhang^1,67^, John R Attia^24^, Adam S Butterworth^27,185^, Daniel I Chasman^23,163^, David Conen^186,187^, Francesco Cucca^188,189^, John Danesh^27,185^, Caroline Hayward^19^, Joanna MM Howson^27^, Markku Laakso^190^, Edward G Lakatta^191^, Claudia Langenberg^28^, Olle Melander^18^, Dennis O Mook-Kanamori^26,192^, Colin NA Palmer^32^, Lorenz Risch^193-195^, Robert A Scott^28^, Rodney J Scott^24^, Peter Sever^128^, Tim D Spector^29^, Pim van der Harst^196^, Nicholas J Wareham^28^, Eleftheria Zeggini^31^, Daniel Levy^107,118^, Patricia B Munroe^3,4^, Christopher Newton-Cheh^134,197,198^, Morris J Brown^3,4^, Andres Metspalu^16^, Bruce M. Psaty^201,202^, Louise V Wain^54^, Paul Elliott^1,5,203-205^, Mark J Caulfield^3,4^

1. Department of Epidemiology and Biostatistics, Imperial College London, London, UK.

2. Department of Hygiene and Epidemiology, University of Ioannina Medical School, Ioannina, Greece.

3. William Harvey Research Institute, Barts and The London School of Medicine and Dentistry, Queen Mary University of London, London, UK.

4. National Institute for Health Research, Barts Cardiovascular Biomedical Research Center, Queen Mary University of London, London, UK.

5. MRC-PHE Centre for Environment and Health, Imperial College London, London, UK.

7. Division of Epidemiology, Department of Medicine, Institute for Medicine and Public Health, Vanderbilt Genetics Institute, Vanderbilt University Medical Center, Tennessee Valley Healthcare System (626)/Vanderbilt University, Nashville, TN, USA.

8. Vanderbilt Genetics Institute, Vanderbilt Epidemiology Center, Department of Obstetrics and Gynecology, Vanderbilt University Medical Center; Tennessee Valley Health Systems VA, Nashville, TN, USA.

9. Department of Epidemiology, Emory University Rollins School of Public Health, Atlanta, GA, USA.

10. Department of Biomedical Informatics, Emory University School of Medicine, Atlanta, GA, USA.

11. Massachusetts Veterans Epidemiology Research and Information Center (MAVERIC), VA Boston Healthcare System, Boston, USA.

12. Division of Aging, Department of Medicine, Brigham and Women’s Hospital, Boston, MA, Department of Medicine, Harvard Medical School, Boston, MA, USA.

13. Atlanta VAMC and Emory Clinical Cardiovascular Research Institute, Atlanta, GA, USA.

14. VA Palo Alto Health Care System; Division of Cardiovascular Medicine, Stanford University School of Medicine, CA, USA.

15. Nephrology Section, Memphis VA Medical Center and University of Tennessee Health Science Center, Memphis, TN, USA.

16. Estonian Genome Center, University of Tartu, Tartu, Estonia.

17. Program in Medical and Population Genetics, Broad Institute of Harvard and MIT, Cambridge, MA, USA.

18. Department Clinical Sciences, Malmö, Lund University, Malmö, Sweden.

19. MRC Human Genetics Unit, MRC Institute of Genetics and Molecular Medicine, University of Edinburgh, Western General Hospital, Edinburgh, Scotland, UK

20. Department of Neurology, Bordeaux University Hospital, Bordeaux, France.

21. Univ. Bordeaux, Inserm, Bordeaux Population Health Research Center, CHU Bordeaux, Bordeaux, France​.

22. Laboratory of Genetics and Genomics, NIA/NIH , Baltimore, MD, USA.

23. Division of Preventive Medicine, Brigham and Women's Hospital, Boston, MA, USA.

24. Hunter Medical Reseach Institute and Faculty of Health, University of Newcastle, New Lambton Heights, New South Wales, Australia.

25. Department of Biostatistics and Center for Statistical Genetics, University of Michigan, Ann Arbor, MI, USA.

26. Department of Clinical Epidemiology, Leiden University Medical Center, Leiden, the Netherlands.

27. MRC/BHF Cardiovascular Epidemiology Unit, Department of Public Health and Primary Care, University of Cambridge, Cambridge, UK.

28. MRC Epidemiology Unit, University of Cambridge School of Clinical Medicine, Cambridge, UK.

29. Department of Twin Research and Genetic Epidemiology, Kings College London, London, UK.

30. NIHR Biomedical Research Centre at Guy’s and St Thomas’ Foundation Trust, London, UK.

31. Wellcome Trust Sanger Institute, Hinxton, UK.

32. Division of Molecular and Clinical Medicine, School of Medicine, University of Dundee, UK.

33. Department of Pharmacy, COMSATS Institute of Information Technology, Abbottabad, Pakistan.

34. Department of Pathology and Molecular Medicine, McMaster University, Hamilton, Canada.

35. Institut universitaire de cardiologie et de pneumologie de Québec-Université Laval, , Quebec City, Canada.

36. Cardiovascular Research Center and Center for Human Genetic Research, Massachusetts General Hospital, Boston, Massachusetts, ΜΑ, USA.

37. University of Groningen, University Medical Center Groningen, Department of Cardiology, Groningen, The Netherlands.

38. University of Lille, Inserm, Centre Hosp. Univ Lille, Institut Pasteur de Lille, UMR1167 - RID-AGE - Risk factors and molecular determinants of aging-related diseases, Epidemiology and Public Health Department, Lille, France.

39. University of Dundee, Ninewells Hospital & Medical School, Dundee, , UK.

40. Wellcome Trust Centre for Human Genetics, University of Oxford, Oxford, UK.

41. Division of Cardiovascular Medicine, Radcliffe Department of Medicine, University of Oxford, Oxford, UK.

42. Usher Institute of Population Health Sciences and Informatics, University of Edinburgh, UK.

43. Department of Internal Medicine, Section Gerontology and Geriatrics, Leiden University Medical Center, Leiden, The Netherlands.

44. Imperial Clinical Trials Unit, Stadium House, 68 Wood Lane, London, UK.

45. School of Medicine, University College Dublin, Ireland.

46. Molecular and Cellular Therapeutics, Royal College of Surgeons in Ireland, Dublin, Ireland.

47. International Centre for Circulatory Health, Imperial College London, London, UK.

48. Center for Statistical Genetics, Dept. of Biostatistics, SPH II, Washington Heights, Ann Arbor, MI, USA.

49. Genetic Epidemiology Unit, Department of Epidemiology, Erasmus MC, Rotterdam, the Netherlands.

50. Center for Complex Disease Genomics, McKusick-Nathans Institute of Genetic Medicine, Johns Hopkins University School of Medicine, Baltimore, MD, USA.

51. Institute of Genetic Medicine, Newcastle University, Newcastle upon Tyne, UK.

52. Sema4, a Mount Sinai venture, Stamford, CT, USA.

53. Division of Genetics and Cell Biology, San Raffaele Scientific Institute, Milano, Italy.

54. Department of Health Sciences, University of Leicester, Leicester, UK.

55. Cardiovascular Health Research Unit, Department of Medicine, University of Washington, Seattle, WA, USA.

56. Institute of Social and Preventive Medicine, University Hospital of Lausanne, Lausanne, Switzerland.

57. Human Genetics Center, School of Public Health, The University of Texas Health Science Center at Houston and Human Genome Sequencing Center, Baylor College of Medicine, One Baylor Plaza, Houston, TX, USA.

58. Department of Biological Psychology, Vrije Universiteit Amsterdam, EMGO+ institute, VU University medical center, Amsterdam, the Netherlands.

59. The Charles Bronfman Institute for Personalized Medicine, Icachn School of Medicine at Mount Sinai, NY, USA.

60. Department of Cardiovascular Sciences, University of Leicester, Leicester, UK.

61.  NIHR Leicester Biomedical Research Centre, Glenfield Hospital, Groby Road, Leicester, UK.

62. Department of Medical, Surgical and Health Sciences, University of Trieste, , Trieste, Italy.

63. Medical Genetics Section, Centre for Genomic and Experimental Medicine, Institute of Genetics and Molecular Medicine, University of Edinburgh, Edinburgh, UK.

64. Generation Scotland, Centre for Genomic and Experimental Medicine, University of Edinburgh, Edinburgh, UK.

65. Centre for Global Health Research, Usher Institute of Population Health Sciences and Informatics, University of Edinburgh, Edinburgh, Scotland, UK

66. Lee Kong Chian School of Medicine, Nanyang Technological University, Singapore, Singapore.

67. Department of Cardiology, Ealing Hospital, Middlesex, UK.

68. Imperial College Healthcare NHS Trust, London, UK.

69. Centre for Brain Research, Indian Institute of Science, Bangalore, India.

70. Institute of Genetics and Biophysics "A. Buzzati-Traverso", CNR, Napoli, Italy.

71. IRCCS Neuromed, Pozzilli, Isernia, Italy.

72. Institute for Maternal and Child Health IRCCS Burlo Garofolo, Trieste, Italy.

73. Medical Genomics and Metabolic Genetics Branch, National Human Genome Research Institute, NIH, Bethesda, MD, USA.

74. Centre for Cognitive Ageing and Cognitive Epidemiology, University of Edinburgh, 7 George Square, Edinburgh, UK.

75. Department of Psychology, University of Edinburgh, 7 George Square, Edinburgh, UK.

76. Department of Internal Medicine, Division of Nephrology, University of Groningen, University Medical Center Groningen, Groningen, The Netherlands.

77. Department of Molecular Epidemiology, Leiden University Medical Center, Leiden, the Netherlands.

78. Institute for Biomedicine, Eurac Research, Bolzano, Italy - Affiliated Institute of the University of Lübeck, Lübeck, Germany.

79. Mathematical and Statistical Computing Laboratory, Office of Intramural Research, Center for Information Technology, National Institutes of Health, Bethesda, MD, USA.

80. Department of Internal Medicine B, University Medicine Greifswald, Greifswald, Germany.

81. DZHK (German Centre for Cardiovascular Research), partner site Greifswald, Greifswald, Germany.

82. Cardiology, Department of Medicine, Geneva University Hospital, Geneva, Switzerland.

83. CIBERCV & Cardiovascular Epidemiology and Genetics, IMIM. Dr Aiguader 88, Barcelona, Spain.

84. Faculty of Medicine, Universitat de Vic-Central de Catalunya, Vic, Spain.

85. Department of Immunology, Genetics and Pathology, Uppsala Universitet, Science for Life Laboratory, Uppsala, Sweden.

86. Wellcome Centre for Human Genetics, University of Oxford, Roosevelt Drive, Oxford, UK.

87. Big Data Institute, Li Ka Shing Center for Health for Health Information and Discovery, Oxford University, Old Road, Oxford, UK.

88. Cardiovascular Medicine Unit, Department of Medicine Solna, Karolinska Institutet, Stockholm, Sweden.

89. Centre for Molecular Medicine, L8:03, Karolinska Universitetsjukhuset, Solna, Sweden.

90. Department of Numerical Analysis and Computer Science, Stockholm University, Stockholm, Sweden.

91. Department of Epidemiology, Erasmus MC, Rotterdam, the Netherlands.

92. Department of Public Health and Caring Sciences, Geriatrics, Uppsala, Sweden.

93. Research Unit of Molecular Epidemiology, Helmholtz Zentrum München, German Research Center for Environmental Health, Neuherberg, Germany.

94. Institute of Epidemiology, Helmholtz Zentrum München, German Research Center for Environmental Health, Neuherberg, Germany.

95. German Center for Diabetes Research (DZD e.V.), Neuherberg, Germany.

96. Department of Psychology, School of Social Sciences, Heriot-Watt University, Edinburgh, UK.

97. Faculty of Medicine, University of Iceland, Reykjavik, Iceland.

98. Icelandic Heart Association, Kopavogur, Iceland.

99. The Institute for Translational Genomics and Population Sciences, Department of Pediatrics, LABioMed at Harbor-UCLA Medical Center, Torrance, CA, USA.

100. Intramural Research Program, Laboratory of Epidemiology, Demography, and Biometry, National Institute on Aging, Bethesda, MD, USA.

101. Department of Psychiatry, University of Groningen, University Medical Center Groningen, Groningen, The Netherlands.

102. Department of Public Health Solutions, National Institute for Health and Welfare (THL), Helsinki, Finland.

103. Institute for Molecular Medicine Finland (FIMM), University of Helsinki, Helsinki, Finland.

104. Clinical Division of Neurogeriatrics, Department of Neurology, Medical University of Graz, Graz, Austria.

105. Institute for Medical Informatics, Statistics and Documentation, Medical University of Graz, Graz, Austria.

106. Department of Epidemiology, Harvard T.H. Chan School of Public Health, Boston, MA, USA.

107. National Heart, Lung and Blood Institute's Framingham Heart Study, Framingham, MA, USA.

108. The Population Science Branch, Division of Intramural Research, National Heart Lung and Blood Institute national Institute of Health, Bethesda, MD, USA.

109. Department of Medical Sciences, Molecular Epidemiology and Science for Life Laboratory, Uppsala University, Uppsala, Sweden.

110. Division of Cardiovascular Medicine, Department of Medicine, Stanford University School of Medicine, Stanford, CA USA.

111. Department of Pulmonary Physiology and Sleep, Sir Charles Gairdner Hospital, Hospital Avenue, Nedlands, Australia.

112. School of Medicine and Pharmacology, University of Western Australia.

113. Department of Psychiatry, VU University Medical Center, Amsterdam Neuroscience, Amsterdam, the Netherlands.

114. Biocenter Oulu, University of Oulu, Oulu, Finland.

115. Center For Life-course Health Research, University of Oulu, Oulu Finland.

116. Unit of Primary Care, Oulu University Hospital, Oulu, Oulu, Finland.

117. Hebrew SeniorLife, Harvard Medical School, Boston, MA, USA.

118. Population Sciences Branch, National Heart, Lung and Blood Institute, National Institutes of Health, Bethesda, MD, USA.

119. Department of Cardiology, Leiden University Medical Center, Leiden, the Netherlands.

120. Department of Clinical Physiology, Tampere University Hospital, Tampere, Finland.

121. Department of Clinical Physiology, Finnish Cardiovascular Research Center - Tampere, Faculty of Medicine and Life Sciences, University of Tampere, Tampere, Finland.

122. Broad Institute of the Massachusetts Institute of Technology and Harvard University, Cambridge, MA, USA.

123. Division of Cardiovascular Sciences, Faculty of Biology, Medicine and Health, The University of Manchester, Manchester, UK.

124. Division of Medicine, Manchester University NHS Foundation Trust, Manchester Academic Health Science Centre, Manchester, UK

125. Department of Public Health and Primary Care, Institute of Public Health, University of Cambridge, Cambridge, UK.

126. Data Science Institute and Lancaster Medical School, Lancaster, UK.

127. Department of Public Health, Faculty of Medicine, University of Split, Croatia.

128. National Heart and Lung Institute, Imperial College London, London, UK.

129. Swiss Institute of Bioinformatics, Lausanne, Switzerland.

130. Institute of Biomedicine and Translational Medicine, University of Tartu, Tartu, Estonia.

131. Department of Clinical Chemistry, Fimlab Laboratories, Tampere, Finland.

132. Department of Clinical Chemistry, Finnish Cardiovascular Research Center - Tampere, Faculty of Medicine and Life Sciences, University of Tampere, Tampere, Finland

133. Department of Medical Sciences, Cardiovascular Epidemiology, Uppsala University, Uppsala, Sweden.

134. Program in Medical and Population Genetics, Broad Institute, Cambridge, MA, USA.

135. Division of Public Health Sciences, Wake Forest School of Medicine, Winston-Salem, NC, USA.

136. Mindich Child health Development Institute, The Icahn School of Medicine at Mount Sinai, New York, NY, USA.

137. Department of Psychiatry, Royal College of Surgeons in Ireland, Education and Research Centre, Beaumont Hospital, Dublin, Ireland.

138. University College Dublin, UCD Conway Institute, Centre for Proteome Research, UCD, Belfield, Dublin, Ireland.

139. Institute of Health and Society, Newcastle University, Newcastle upon Tyne, UK.

140. Department of Psychiatry, Amsterdam Public Health and Amsterdam Neuroscience, VU University Medical Center/GGZ inGeest, Amsterdam, The Netherlands.

141. Department of Biostatistics, University of Liverpool, Block F, Waterhouse Building, Liverpool, UK.

142. Department of Epidemiology, Human Genetics and Environmental Sciences, School of Public Health, University of Texas Health Science Center at Houston, Houston, TX, USA.

143. Data Tecnica International, Glen Echo, MD, USA.

144. Laboratory of Neurogenetics, National Institute on Aging, Bethesda, USA.

145. Department of Medicine, Turku University Hospital and University of Turku, Finland.

146. Department of Epidemiology, University of Groningen, University Medical Center Groningen, Groningen, The Netherlands.

147. Interdisciplinary Center Psychopathology and Emotion regulation (ICPE), University of Groningen, University Medical Center Groningen, Groningen, The Netherlands.

148. SGDP Centre, Institute of Psychiatry, Psychology and Neuroscience, King's College London, London, UK.

149. British Heart Foundation Glasgow Cardiovascular Research Centre, Institute of Cardiovascular and Medical Sciences, College of Medical, Veterinary and Life Sciences, University of Glasgow, Glasgow, UK.

150. Department of Medicine, Columbia University Medical Center, New York, NY, USA.

151. Analytic and Translational Genetics Unit, Department of Medicine, Department of Neurology and Department of Psychiatry Massachusetts General Hospital, Boston, MA, USA.

152. The Stanley Center for Psychiatric Research and Program in Medical and Population Genetics, The Broad Institute of MIT and Harvard, Cambridge, MA, USA.

153. University of Tartu, Tartu, Estonia.

154. German Center for Cardiovascular Disease Research (DZHK), partner site Munich, Neuherberg, Germany.

155. Psychiatric hospital “Sveti Ivan”, Zagreb, Croatia.

156. Department of Neurology, General Central Hospital, Bolzano, Italy.

157. Department of Neurology, University of Lübeck, Lübeck, Germany.

158. Department of Clinical Physiology and Nuclear Medicine, Turku University Hospital, Turku, Finland.

159. Research Centre of Applied and Preventive Cardiovascular Medicine, University of Turku, Turku, Finland.

161. Institute of Physiology, University Medicine Greifswald, Karlsburg, Germany.

162. Department of Biostatistics University of Washington, Seattle, WA, USA.

163. Harvard Medical School, Boston MA.

164. Public health, Faculty of Medicine, University of Helsinki, Finland

165. Centre for Global Health Research, Usher Institute of Population Health Sciences and Informatics, University of Edinburgh, Scotland, UK.

166. Gottfried Schatz Research Center for Cell Signaling, Metabolism & Aging, Molecular Biology and Biochemistry, Medical University of Graz, Graz, Austria.

167. The New York Academy of Medicine, New York, NY, USA.

168. Alzheimer Scotland Dementia Research Centre, University of Edinburgh, Edinburgh, UK.

169. Institute of Cardiovascular and Medical Sciences, Faculty of Medicine, University of Glasgow, United Kingdom.

170. Population Health Research Institute, St George's, University of London, London, UK.

171. Department of Genetics, University of Groningen, University Medical Center Groningen, Groningen, The Netherlands.

172. Institute for Community Medicine, University Medicine Greifswald, Greifswald, Germany.

173. Department of Gerontology and Geriatrics, Leiden University Medical Center, Leiden, the Netherlands.

174. Dasman Diabetes Institute, Dasman, Kuwait.

175. Chronic Disease Prevention Unit, National Institute for Health and Welfare, Helsinki, Finland.

176. Department of Public Health, University of Helsinki, Helsinki, Finland.

177. Saudi Diabetes Research Group, King Abdulaziz University, Jeddah, Saudi Arabia.

178. Department of Internal Medicine, Erasmus MC, Rotterdam, the Netherlands.

179. Research Institute for Primordial Prevention of Non-communicable Disease, Isfahan University of Medical Sciences, Isfahan, Iran.

180. Interfaculty Institute for Genetics and Functional Genomics, University Medicine Greifswald, Greifswald, Germany.

181. Department of Internal Medicine, University Hospital, CHUV, Lausanne, Switzerland.

182. Experimental Genetics Division, Sidra Medical and Research Center, Doha, Qatar.

183. Centre for Population Health Sciences, Usher Institute of Population Health Sciences and Informatics, University of Edinburgh, Scotland, UK

184. Department of Biology, Faculty of Medicine, University of Split, Croatia.

185. The National Institute for Health Research Blood and Transplant Research Unit in Donor Health and Genomics, University of Cambridge, UK.

186. Division of Cardiology, University Hospital, Basel, Switzerland.

187. Division of Cardiology, Department of Medicine, McMaster University, Hamilton, Canada.

188. Institute of Genetic and Biomedical Research, National Research Council (CNR), Monserrato, Cagliari, Italy.

189. Department of Biomedical Sciences, University of Sassari, Sassari, Italy.

190. Institute of Clinical Medicine, Internal Medicine, University of Eastern Finland and Kuopio University Hospital, Kuopio, Finland.

191. Laboratory of Cardiovascular Science, NIA/NIH , Baltimore, MD, USA.

192. Department of Public Health and Primary Care, Leiden University Medical Center, Leiden, the Netherlands.

193. Labormedizinisches Zentrum Dr. Risch, Schaan, Liechtenstein.

194. Private University of the Principality of Liechtenstin, Triesen, Liechtenstein.

195. University Insitute of Clinical Chemistry, Inselspital, Bern University Hospital, University of Bern, Bern, Switzerland.

196. Department of Cardiology, University of Groningen, University Medical Center Groningen, Groningen, The Netherlands.

197. Center for Genomic Medicine, Massachusetts General Hospital, Boston, MA, USA.

198. Cardiovascular Research Center, Massachusetts General Hospital, Boston, MA, USA.

201. Cardiovascular Health Research Unit, Departments of Medicine, Epidemiology and Health Services, University of Washington, Seattle, WA, USA.

202. Kaiser Permanente Washington Health Research Institute, Seattle, WA, USA.

203. National Institute for Health Research Imperial Biomedical Research Centre, Imperial College Healthcare NHS Trust and Imperial College London, London, UK.

204. UK Dementia Research Institute (UK DRI) at Imperial College London, London, UK

205. Health Data Research-UK London substantive site, London, U.K

**Supplementary Document 2. STROBE-MR checklist of recommended items to address in reports of Mendelian Randomization studies**

**STROBE-MR checklist of recommended items to address in reports of Mendelian randomization studies**^1-2^

| **Item** | **Section** | **Checklist item** |
| --- | --- | --- |
| **No.** |  |  |
|  |  |  |
| 1 | **TITLE and** | Indicate Mendelian randomization (MR) as the study’s design in the title and/or the |
|  | **ABSTRACT** | abstract if that is a main purpose of the study |
|  |  |  |
|  | **INTRODUCTION** |  |
|  |  |  |
| 2 | **Background** | Explain the scientific background and rationale for the reported study. What is the |
|  |  | exposure? Is a potential causal relationship between exposure and outcome |
|  |  | plausible? Justify why MR is a helpful method to address the study question |
|  |  |  |
| 3 | **Objectives** | State specific objectives clearly, including pre-specified causal hypotheses (if any). |
|  |  | State that MR is a method that, under specific assumptions, intends to estimate |
|  |  | causal effects |
|  |  |  |
|  | **METHODS** |  |
|  |  |  |
| 4 | **Study design and** | Present key elements of the study design early in the article. Consider including a |
|  | **data sources** | table listing sources of data for all phases of the study. For each data source |

contributing to the analysis, describe the following:

1. Setting: Describe the study design and the underlying population, if possible. Describe the setting, locations, and relevant dates, including periods of recruitment, exposure, follow-up, and data collection, when available.
2. Participants: Give the eligibility criteria, and the sources and methods of selection of participants. Report the sample size, and whether any power or sample size calculations were carried out prior to the main analysis
3. Describe measurement, quality control and selection of genetic variants
4. For each exposure, outcome, and other relevant variables, describe methods of assessment and diagnostic criteria for diseases
5. Provide details of ethics committee approval and participant informed consent, if relevant

| 5 | **Assumptions** | Explicitly state the three core IV assumptions for the main analysis (relevance, |
| --- | --- | --- |
|  |  | independence and exclusion restriction) as well assumptions for any additional or |
|  |  | sensitivity analysis |

1. **Statistical methods:** Describe statistical methods and statistics used

**main analysis**

- 1. Describe how quantitative variables were handled in the analyses (i.e., scale, units, model)
  2. Describe how genetic variants were handled in the analyses and, if applicable, how their weights were selected
  3. Describe the MR estimator (e.g., two-stage least squares, Wald ratio) and related statistics. Detail the included covariates and, in case of two-sample MR, whether the same covariate set was used for adjustment in the two samples

d) Explain how missing data were addressed

1. If applicable, indicate how multiple testing was addressed

| 7 | **Assessment of** | Describe any methods or prior knowledge used to assess the assumptions or justify |
| --- | --- | --- |
|  | **assumptions** | their validity |
|  |  |  |
| 8 | **Sensitivity analyses** | Describe any sensitivity analyses or additional analyses performed (e.g., comparison |
|  | **and additional** | of effect estimates from different approaches, independent replication, bias analytic |
|  | **analyses** | techniques, validation of instruments, simulations) |

1. **Software and pre-**

**registration**

1. Name statistical software and package(s), including version and settings used
2. State whether the study protocol and details were pre-registered (as well as when and where)

**RESULTS**

10 **Descriptive data**

1. Report the numbers of individuals at each stage of included studies and reasons for exclusion. Consider use of a flow diagram
2. Report summary statistics for phenotypic exposure(s), outcome(s), and other relevant variables (e.g., means, SDs, proportions)
3. If the data sources include meta-analyses of previous studies, provide the assessments of heterogeneity across these studies
4. For two-sample MR:
   1. Provide justification of the similarity of the genetic variant-exposure associations between the exposure and outcome samples
   2. Provide information on the number of individuals who overlap between the exposure and outcome studies
5. **Main results**
6. Report the associations between genetic variant and exposure, and between genetic variant and outcome, preferably on an interpretable scale
7. Report MR estimates of the relationship between exposure and outcome, and the measures of uncertainty from the MR analysis, on an interpretable scale, such as odds ratio or relative risk per SD difference
8. If relevant, consider translating estimates of relative risk into absolute risk for a meaningful time period
9. Consider plots to visualize results (e.g., forest plot, scatterplot of associations between genetic variants and outcome versus between genetic variants and exposure)
10. **Assessment of assumptions**
11. Report the assessment of the validity of the assumptions
12. Report any additional statistics (e.g., assessments of heterogeneity across genetic variants, such as *I^2^*, Q statistic or E-value)
13. **Sensitivity analyses and additional analyses**

|  | a) | Report any sensitivity analyses to assess the robustness of the main results to |
| --- | --- | --- |
|  |  | violations of the assumptions |
|  | b) | Report results from other sensitivity analyses or additional analyses |
|  | c) | Report any assessment of direction of causal relationship (e.g., bidirectional MR) |
|  | d) | When relevant, report and compare with estimates from non-MR analyses |
|  | e) | Consider additional plots to visualize results (e.g., leave-one-out analyses) |
|  | **DISCUSSION** |  |
| 14 | **Key results** | Summarize key results with reference to study objectives |
| 15 | **Limitations** | Discuss limitations of the study, taking into account the validity of the IV assumptions, |
|  |  | other sources of potential bias, and imprecision. Discuss both direction and |
|  |  | magnitude of any potential bias and any efforts to address them |

16 **Interpretation**

1. Meaning: Give a cautious overall interpretation of results in the context of their limitations and in comparison with other studies
2. Mechanism: Discuss underlying biological mechanisms that could drive a potential causal relationship between the investigated exposure and the outcome, and whether the gene-environment equivalence assumption is reasonable. Use causal language carefully, clarifying that IV estimates may provide causal effects only under certain assumptions
3. Clinical relevance: Discuss whether the results have clinical or public policy relevance, and to what extent they inform effect sizes of possible interventions

|  | 17 | **Generalizability** | Discuss the generalizability of the study results (a) to other populations, (b) across |
| --- | --- | --- | --- |
|  |  |  | other exposure periods/timings, and (c) across other levels of exposure |
|  |  | **OTHER** |  |
|  |  | **INFORMATION** |  |
|  | 18 | **Funding** | Describe sources of funding and the role of funders in the present study and, if |
|  |  |  | applicable, sources of funding for the databases and original study or studies on |
|  |  |  | which the present study is based |
|  | 19 | **Data and data** | Provide the data used to perform all analyses or report where and how the data can |
|  |  | **sharing** | be accessed, and reference these sources in the article. Provide the statistical code |
|  |  |  | needed to reproduce the results in the article, or report whether the code is publicly |
|  |  |  | accessible and if so, where |
|  | 20 | **Conflicts of** | All authors should declare all potential conflicts of interest |
|  |  | **Interest** |  |

This checklist is copyrighted by the Equator Network under the Creative Commons Attribution 3.0 Unported (CC BY 3.0) license.

1. Skrivankova VW, Richmond RC, Woolf BAR, Yarmolinsky J, Davies NM, Swanson SA, et al. Strengthening the Reporting of Observational Studies in Epidemiology using Mendelian Randomization (STROBE-MR) Statement. JAMA. 2021;326(16):1614-1621.
2. Skrivankova VW, Richmond RC, Woolf BAR, Davies NM, Swanson SA, VanderWeele TJ, et al. Strengthening the Reporting of Observational Studies in Epidemiology using Mendelian Randomisation (STROBE-MR): Explanation and Elaboration. BMJ. 2021;375:n2233.

**Supplementary References**

1. Sudlow C, Gallacher J, Allen N, Beral V, Burton P, Danesh J, et al. Uk Biobank: An Open Access Resource for Identifying the Causes of a Wide Range of Complex Diseases of Middle and Old Age. *PLoS Med* (2015) 12(3):e1001779. Epub 2015/04/01. doi: 10.1371/journal.pmed.1001779.

2. Sijtsma A, Rienks J, van der Harst P, Navis G, Rosmalen JGM, Dotinga A. Cohort Profile Update: Lifelines, a Three-Generation Cohort Study and Biobank. *Int J Epidemiol* (2021). Epub 2021/12/14. doi: 10.1093/ije/dyab257.

3. Verweij N, van de Vegte YJ, van der Harst P. Genetic Study Links Components of the Autonomous Nervous System to Heart-Rate Profile During Exercise. *Nat Commun* (2018) 9(1):898. Epub 2018/03/03. doi: 10.1038/s41467-018-03395-6.

4. Uk Biobank Cardio Assessment V.1.0. (Uk Biobank, 2011). Available from: https://biobank.ctsu.ox.ac.uk/crystal/ukb/docs/Cardio.pdf.

5. Tegegne BS, Man T, van Roon AM, Riese H, Snieder H. Determinants of Heart Rate Variability in the General Population: The Lifelines Cohort Study. *Heart Rhythm* (2018) 15(10):1552-8. Epub 2018/05/13. doi: 10.1016/j.hrthm.2018.05.006.

6. van Roon AM, Snieder H, Lefrandt JD, de Geus EJ, Riese H. Parsimonious Correction of Heart Rate Variability for Its Dependency on Heart Rate. *Hypertension* (2016) 68(5):e63-e5. Epub 2016/09/28. doi: 10.1161/HYPERTENSIONAHA.116.08053.

7. Tobin MD, Sheehan NA, Scurrah KJ, Burton PR. Adjusting for Treatment Effects in Studies of Quantitative Traits: Antihypertensive Therapy and Systolic Blood Pressure. *Stat Med* (2005) 24(19):2911-35. Epub 2005/09/10. doi: 10.1002/sim.2165.

8. Said MA, Eppinga RN, Lipsic E, Verweij N, van der Harst P. Relationship of Arterial Stiffness Index and Pulse Pressure with Cardiovascular Disease and Mortality. *J Am Heart Assoc* (2018) 7(2). Epub 2018/01/24. doi: 10.1161/JAHA.117.007621.

9. van der Ende MY, Hartman MH, Hagemeijer Y, Meems LM, de Vries HS, Stolk RP, et al. The Lifelines Cohort Study: Prevalence and Treatment of Cardiovascular Disease and Risk Factors. *Int J Cardiol* (2017) 228:495-500. Epub 2016/11/23. doi: 10.1016/j.ijcard.2016.11.061.

10. van Zon SKR, Reijneveld SA, van der Most PJ, Swertz MA, Bultmann U, Snieder H. The Interaction of Genetic Predisposition and Socioeconomic Position with Type 2 Diabetes Mellitus: Cross-Sectional and Longitudinal Analyses from the Lifelines Cohort and Biobank Study. *Psychosom Med* (2018) 80(3):252-62. Epub 2018/01/31. doi: 10.1097/PSY.0000000000000562.

11. Groot HE, van de Vegte YJ, Verweij N, Lipsic E, Karper JC, van der Harst P. Human Genetic Determinants of the Gut Microbiome and Their Associations with Health and Disease: A Phenome-Wide Association Study. *Sci Rep* (2020) 10(1):14771. Epub 2020/09/10. doi: 10.1038/s41598-020-70724-5.

12. Unger T, Borghi C, Charchar F, Khan NA, Poulter NR, Prabhakaran D, et al. 2020 International Society of Hypertension Global Hypertension Practice Guidelines. *J Hypertens* (2020) 38(6):982-1004. Epub 2020/05/07. doi: 10.1097/HJH.0000000000002453.

13. Tegegne B. The Heart of the Matter: Discovery of New Genetic Loci for Heart Rate Variability and Its Relationship with Blood Pressure and Mortality.: University of Groningen (2021).

14. Uk Biobank Gwas Results. Available from: http://www.nealelab.is/uk-biobank/.

15. Evangelou E, Warren HR, Mosen-Ansorena D, Mifsud B, Pazoki R, Gao H, et al. Genetic Analysis of over 1 Million People Identifies 535 New Loci Associated with Blood Pressure Traits. *Nat Genet* (2018) 50(10):1412-25. doi: 10.1038/s41588-018-0205-x.

16. Siedlinski M, Jozefczuk E, Xu X, Teumer A, Evangelou E, Schnabel RB, et al. White Blood Cells and Blood Pressure: A Mendelian Randomization Study. *Circulation* (2020) 141(16):1307-17. Epub 2020/03/10. doi: 10.1161/CIRCULATIONAHA.119.045102.

17. Hemani G, Tilling K, Davey Smith G. Orienting the Causal Relationship between Imprecisely Measured Traits Using Gwas Summary Data. *PLoS Genet* (2017) 13(11):e1007081. Epub 2017/11/18. doi: 10.1371/journal.pgen.1007081.

18. Burgess S, Bowden J, Fall T, Ingelsson E, Thompson SG. Sensitivity Analyses for Robust Causal Inference from Mendelian Randomization Analyses with Multiple Genetic Variants. *Epidemiology* (2017) 28(1):30-42. Epub 2016/10/18. doi: 10.1097/EDE.0000000000000559.

19. Bowden J, Davey Smith G, Haycock PC, Burgess S. Consistent Estimation in Mendelian Randomization with Some Invalid Instruments Using a Weighted Median Estimator. *Genet Epidemiol* (2016) 40(4):304-14. Epub 2016/04/12. doi: 10.1002/gepi.21965.

20. Bowden J, Davey Smith G, Burgess S. Mendelian Randomization with Invalid Instruments: Effect Estimation and Bias Detection through Egger Regression. *Int J Epidemiol* (2015) 44(2):512-25. Epub 2015/06/08. doi: 10.1093/ije/dyv080.

21. Haycock PC, Burgess S, Wade KH, Bowden J, Relton C, Davey Smith G. Best (but Oft-Forgotten) Practices: The Design, Analysis, and Interpretation of Mendelian Randomization Studies. *Am J Clin Nutr* (2016) 103(4):965-78. Epub 2016/03/11. doi: 10.3945/ajcn.115.118216.

22. Hemani G, Bowden J, Davey Smith G. Evaluating the Potential Role of Pleiotropy in Mendelian Randomization Studies. *Hum Mol Genet* (2018) 27(R2):R195-R208. Epub 2018/05/18. doi: 10.1093/hmg/ddy163.

23. Burgess S, Davies NM, Thompson SG. Bias Due to Participant Overlap in Two-Sample Mendelian Randomization. *Genet Epidemiol* (2016) 40(7):597-608. Epub 2016/10/19. doi: 10.1002/gepi.21998.

24. Munafo MR, Tilling K, Taylor AE, Evans DM, Davey Smith G. Collider Scope: When Selection Bias Can Substantially Influence Observed Associations. *Int J Epidemiol* (2018) 47(1):226-35. Epub 2017/10/19. doi: 10.1093/ije/dyx206.

25. Gilbody J, Borges MC, Smith GD, Sanderson E. Multivariable Mr Can Mitigate Bias in Two-Sample Mr Using Covariable-Adjusted Summary Associations. (2022):2022.07.19.22277803. doi: 10.1101/2022.07.19.22277803 %J medRxiv.

26. Burgess S, Thompson SG, Collaboration CCG. Avoiding Bias from Weak Instruments in Mendelian Randomization Studies. *Int J Epidemiol* (2011) 40(3):755-64. Epub 2011/03/19. doi: 10.1093/ije/dyr036.

27. Bowden J, Del Greco MF, Minelli C, Davey Smith G, Sheehan NA, Thompson JR. Assessing the Suitability of Summary Data for Two-Sample Mendelian Randomization Analyses Using Mr-Egger Regression: The Role of the I2 Statistic. *Int J Epidemiol* (2016) 45(6):1961-74. Epub 2016/09/13. doi: 10.1093/ije/dyw220.
